# Association between air pollution (PM_10_, PM_2.5_), greenness and depression in older adults: a longitudinal study in South Korea

**DOI:** 10.1017/S2045796024000684

**Published:** 2024-11-27

**Authors:** H. Park, C. Kang, AiMS-CREATE Team, H. Kim

**Affiliations:** 1 Department of Public Health Sciences, Graduate School of Public Health, Seoul National University, Seoul, Republic of Korea; 2 National Evidence-Based Health Care Collaborating Agency, Division of New Health Technology Assessment, Seoul, Republic of Korea; 3 School of Biomedical Convergence Engineering, College of Information and Biomedical Engineering, Pusan National University, Gyeongsangnam-do, Republic of Korea; 4 Department of Environmental Medicine, College of Medicine, Ewha Womans University, Seoul, Republic of Korea; 5 Institute for of Sustainable Development, Seoul National University, Seoul, Republic of Korea

**Keywords:** air pollution, depression, greenness, older Korean adults, NDVI, particulate matter

## Abstract

**Aims:**

Although it has been hypothesized that air pollution, particularly PM_2.5_ and PM_10_, causes depressed symptoms, their interactions with greenness have not yet been confirmed. This study examined the association between depression symptoms and air pollution, as well as the potential moderating effects of greenness.

**Methods:**

A total of 7657 people from all around South Korea were examined using information from the Korean Longitudinal Study of Aging, for the years 2016, 2018 and 2020. Depressive symptoms were assessed using the CES-D 10 score (Center for Epidemiology Studies of Depression scale, Boston form), and annual air pollution levels (PM_2.5_, PM_10_) and greenness (NDVI, Landsat Normalized Difference Vegetation Index) at the district level (si-gun-gu) were considered for the association analysis. The investigation was primarily concerned with determining how the CES-D 10 score changed for each 10 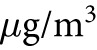 increase in PM_2.5_ and PM_10_ according to NDVI quantiles, respectively. The analysis used generalized estimating equation models that were adjusted with both minimal and complete variables. Subgroup analyses were conducted based on age groups (<65, ≥65 years old), sex and exercise status.

**Results:**

The impact of PM_10_ on depression in the fourth quantile of NDVI was substantially less in the fully adjusted linear mixed model (OR for depression with a 10 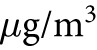 increment of PM_10_: 1.29, 95% CI: 1.06, 1.58) than in the first quantile (OR: 1.88, 95% CI: 1.58, 2.25). In a similar vein, the effect of PM_2.5_ on depression was considerably reduced in the fourth quantile of NDVI (OR for depression with a 10 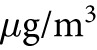 increment of PM_2.5_: 1.78, 95% CI: 1.30, 2.44) compared to the first (OR: 3.75, 95% CI: 2.75, 5.10). Subgroup analysis results demonstrated beneficial effects of greenness in the relationship between particulate matter and depression.

**Conclusions:**

This longitudinal panel study found that a higher quantile of NDVI was associated with a significantly reduced influence of air pollution (PM_10_, PM_2.5_) on depression among older individuals in South Korea.

## Introduction

The International Agency for Research on Cancer classified particulate matter as a Group 1 carcinogen in 2013 (Hamra *et al.*, 2014; IARC, 2015). Particulate matter’s small size allows it to penetrate the brain, contributing to respiratory diseases, cardiovascular disease, diabetes and mental illnesses (Block and Calderón-Garcidueñas, 2009; Calderón-Garcidueñas *et al.*, 2015). Green spaces have been proposed as a way to mitigate the harmful impacts of particulate matter. These spaces offer the potential to mitigate particulate matter through various mechanisms, such as adsorption, blocking and precipitation (Bernatzky, 1983; Choi *et al.*, 2022; Janhäll, 2015; Jin *et al.*, 2014; Liu *et al.*, 2016; Sgrigna *et al.*, 2015; Vesala *et al.*, 2005). The theory of biophillia suggests that green spaces provide benefits by promoting social interaction and social cohesion, which are associated with mental health (White and Heerwagen, 1998; Wilson, 1984). The presence of green spaces, acting as buffers, may mitigate the adverse effects of air pollution on mental health by improving air quality and providing psychological benefits (Gascon *et al.*, 2016; James *et al.*, 2015).

Depression is the most common mental disorder globally and could lead to suicide if it worsens, according to the WHO (WHO, 2023). A recent epidemiological study on mental health revealed that depression prevalence has been gradually rising from 4.0% in 2001 to 6.7% in 2011 in Korea as well, where it has also emerged as a significant health issue (Koo, 2018; Lee and Park, 2014). From 2000 to 2010, the disability-adjusted life years per 100,000 people with major depressive disorder in Korea rose from 427 to 1508 (Lee and Park, 2014). Furthermore, studies suggested a depression prevalence of 26% among individuals aged 60–64 (Park *et al.*, 2013), which increases to an estimated 35.4% among those older than 80 (Park and Kim, 2011). Specifically, in 2014, 46.3% of older adults in South Korea living in relative poverty experienced depression (Kim *et al.*, 2022). The impact of depression might also be underestimated because it is more likely for patients with depression to perform poorly in school, have unstable marriages and relationships, have reduced birth outcomes, experience unemployment, and even have suicidal thoughts, all of which further reduce their quality of life (Kessler, 2012; Ribeiro *et al.*, 2018).

Given the high prevalence and severity of depression, finding modifiable environmental features such as air pollution and greenness cannot be disregarded in the quest to better handle depression. Some epidemiological studies imply that air pollution may be related to mental health, including depression (Braithwaite *et al.*, 2019), while negative associations have been shown between greenness and self-perceived stress (Banay *et al.*, 2019; Roe *et al.*, 2013) or mental health (Gascon *et al.*, 2018). In a longitudinal paper that studied depression, residential greenness and particulate matter, it was found that the protective effect of residential green space was partially mediated by PM_2.5_ and PM_10_ (Zhang *et al.*, 2022). Previous studies focused on the effects of each factor of air pollution and green space or on the effects of simultaneous exposure on depression, and the results of studies on the interaction of the two variables were not sufficient, limiting the interpretation of the study results.

This study used a longitudinal data (Korean Longitudinal Study of Aging [KloSA]) to examine the relationship between air pollution and depressed symptoms as well as how greenness was affected.

## Method

### Population

This study was based on the KLoSA, which was conducted by the Ministry of Employment and the Korea Employment Information Service. Using the stratified systematic sampling method, population survey districts stratified by 15 metropolitan cities, 2 provinces (dong and eup/myeon) and 2 housing types (house and apartment) were arranged by administrative code, and the assigned number of survey districts was extracted by the systematic sampling method. The interviewers visited the extracted households, selected the people aged 45 or older among the household members, and then conducted the Population Research Panel Survey through a computer-assisted personal interview. The KLoSA, which has been conducted every other year since 2006, is a longitudinal survey of the older adults residing in the Korea regions excluding islands, and the eighth survey was finished in 2020. The main purpose of the KLoSA was to produce basic information on older people, including their socioeconomic characteristics, family relationships, living area, psychological health, and health status. The number of participants in local communities nationwide was 10,254 in 2006, and 920 more were registered in 2014. A detailed user’s guide is available on the website (https://www.Klosa.re.kr).

In this analysis, only three waves were used: the sixth wave (*n* = 7490), the seventh wave (*n* = 6940) and the eighth wave (*n* = 6488). The overall weighted response rate was 79.6%, 78.8% and 78.1% for waves 6, 7 and 8, respectively. Among them, those who didn’t complete the questionnaire on depression or socioeconomic characteristics were excluded. The total study population was 7376, 6730 and 6404 in 2016, 2018 and 2020, respectively.

### CES-D 10 (Centre for Epidemiological Studies Depression Scale)

Individual-level time-variant depression was assessed using the CES-D 10 of the Boston Form (Bae *et al.*, 2020; Radloff, 1977). The CES-D 10 is composed of 10 different items from the original 20 (Lewinsohn *et al.*, 1997) and is used to assess respondents’ recent (the past week or so) depression symptoms on a 4-point Likert scale with a cut-off of 20, especially in the elderly population, while the total score of CES-D 10 ranges from 10 to 40, with higher scores indicating more severe depressive symptoms (Lee and Lee, 2014; Pun *et al.*, 2018; Zhang *et al.*, 2012). The questionnaire includes three questions about depressed affect, two questions about positive affect, three questions about somatic symptoms, and two questions about interpersonal relationships. The CES-D 10 exhibited strong internal consistency, as indicated by Cronbach’s alpha coefficients of 0.87, 0.89 and 0.87 in 2016, 2018 and 2020, respectively.

### Air pollution data

We employed monthly concentration predictions for ambient levels of PM_2.5_ and PM_10_ at 1 km^2^ spatial resolution from a machine-learning-based validated model with high prediction performance across 226 districts in contiguous South Korea (cross-validated *R*^2^: 0.87). This predicted air pollution concentration was previously used in a previous paper (Park *et al.*, 2023). The prediction model is described in detail in the supplementary material (see Appendix, 1. Air Pollution Prediction Model). We estimated the annual mean concentration of PM_2.5_ and PM_10_ for each of the 217 districts using this data by averaging the concentrations of grids with the centroid point inside the district boundary. Annual district-specific air pollution concentrations for the years 2016, 2018 and 2020 are then linked to the individuals, representing long-term air pollution exposure levels corresponding to their residential district each year. We also calculated annual concentrations of NO_2_ and 8-hour maximum O_3_ (cross-validated *R*^2^: 0.76 and 0.84, respectively) to utilize other pollutants as confounders to identify the PM_10_ and PM_2.5_ effects after controlling for them.

### Greenness data

The level of residential greenness was assessed by two satellite-image-based metrics: Moderate Resolution Imaging Spectroradiometer (MODIS) normalized difference vegetation index (NDVI) and Enhanced Vegetation Index (EVI). The data, collected at 16-day intervals on a 250 m grid unit basis, involved averaging NDVI and EVI values within each district (si/gun/gu) polygon, utilizing the district shape file. We acquired the datasets from the latest version of the Tropical Rainfall Measuring Mission (TRMM) (https://developers.google.com/earth-engine/datasets/catalog/MODIS_061_MOD13A2, accessed on 8 October 2022) covering the period from 2016 to 2020 for the catchment area. A detailed description is available on the website.

NDVI calculates the ratio of the difference of near-infrared region and red reflectance to the sum of these two and ranges from −1 to +1 (Song *et al.*, 2019). The estimate of EVI is based on NDVI and further controls canopy background, aerosol resistance and gain factor, which means it functions better in densely vegetated areas (Nagler *et al.*, 2013) and has higher sensitivity over high biomass regions. High NDVI and EVI indicate high greenness. The time-varying annual NDVI and EVI of 2016–2020 were matched to the panel data of the same period. The core model and description were based on the NDVI to study the beneficial effect of greenness.

### Controlled variables

Controlled variables were used in this data set, including socioeconomic characteristics and physical condition. Sex was coded as a two-factor variable (female; male). Age was coded as a continuous variable (years). Current smoking was coded as a two-factor variable (no or yes in the past; yes). Current drinking was coded as a two-factor variable (no or yes in the past; yes). Education attainment was coded as a three-factor variable (primary school and below; middle and high school; college and above). Marital status was coded as a two-factor variable (single, divorced and widowed, married and not living with spouse; married and living with spouse). Social contact was coded as a three-factor variable (every other month or less often; once a month or more often and less than once a week; once a week or more often). Self-reported health status was coded as a two-factor variable (normal or bad; good). Private medical insurance was coded as a two-factor variable (no medical insurance; having medical insurance). Exercise was coded as a two-factor variable (no; yes). Household net worth (10000 Korean won, 7.59 USD) was coded as a five-factor variable (∼7000; 7001–15000; 15001–23000; 23001–39000; 39001∼). Density of population (/km^2^) was coded as a five-factor variable (∼280.4; 280.5–1125.5; 1125.6–4745.7; 4745.8–10891.2; 10891.3∼). Longitude, latitude, number of beds in hospitals per 1,000 persons, number of national basic livelihood beneficiaries, independent rate of finance of local government, and proportion of basic pension beneficiaries were coded as continuous variables. We chose covariates to account for possible confounding variables. As basic demographic information, age and sex, behavioural factors such as current smoking, current drinking, socio-economic factors such as private medical insurance, household net worth, education attainment and marital status, known to be closely related to depression were selected (Kim *et al.*, 2020, 2016; Shi *et al.*, 2020; Zhang *et al.*, 2019). Social contact, exercise (Wang *et al.*, 2019b, 2020), self-reported health status (Shi *et al.*, 2020) are important confounders potentially affecting to particulate matters, greenness, and depression, and were also considered. Lastly, we selected regional factors (density of population, number of beds in hospitals per 1,000 persons, number of national basic livelihood beneficiaries, independent rate of finance of local government, proportion of basic pension beneficiaries) because there is evidence that the impact of greenness differs by urbanicity (Huang *et al.*, 2021). We summarised potential confounding factors from the literature using a directed acyclic graph (Supplementary Figure S1).

### Statistical analysis

The study involved the categorization of participants into four groups based on the quantiles of NDVI due to ease of epidemiological interpretation, given the heavily skewed distribution (Banay *et al.*, 2019; White *et al.*, 2021). Descriptive statistics were used to present continuous variables as mean and standard deviation across the four groups, while categorical variables were presented as frequency and proportion. Statistical tests such as Pearson’s chi-squared test and ANOVA were employed to compare categorical and continuous variables, respectively.

To investigate the association between long-term exposure to air pollution (specifically PM_2.5_ and PM_10_) and the score of CES-D 10, as well as the potential protective impact of NDVI on air pollution, generalized estimating equation (GEE) models were utilized. This approach allowed us to analyse longitudinally measured data with binary outcomes, accommodating within-subject variability and changes in outcome status over time within the model. Air pollution variables were treated as continuous, CES-D 10 scores as dichotomous (depression: ≥20, without depression: <20) (Fu *et al.*, 2022; Lee and Lee, 2014), and NDVI as a four-factor variable. The particulate matter data were adjusted for 10 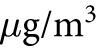. Odds ratios (ORs) and 95% confidence intervals were calculated and reported. The epidemiological perspective would find the OR of depression linked to air pollution more comprehensible. Additionally, interaction effects were examined to identify the potential beneficial effects of NDVI on depression. Interaction terms of NDVI quantiles and particulate matter were included in two exposure models to explore the interaction effects of combined exposures.

The primary hypothesis of the study posited that NDVI would mitigate the adverse impacts of air pollution on depressive symptoms. Three models were employed: an unadjusted model that considered particulate matter (PM_10_ or PM_2.5_), NDVI quantiles, and interaction term of particulate matter and NDVI quantiles; a minimally adjusted model that considered particulate matter, NDVI quantiles, and interaction term of particulate matter and NDVI quantiles, adjusted for year, longitude, latitude, interaction term of longitude and latitude; and a fully adjusted model that considered particulate matter, NDVI quantiles, and interaction term of particulate matter and NDVI quantiles, adjusted for year, longitude, latitude, interaction term of longitude and latitude, age, sex, current smoking, current drinking, education attainment, marital status, social contact, self-reported health status, exercise, private medical insurance, density of population quintiles, number of beds in hospitals per 1,000 persons, number of national basic livelihood beneficiaries, independent rate of finance of local government, proportion of basic pension beneficiaries.

Thus, the stratified group analyses (based on sex, age and exercise) were conducted, revealing that women (Park *et al.*, 2024; Zhang *et al.*, 2019), elderly individuals (Liu *et al.*, 2021), and those with low exercise levels (Zhang *et al.*, 2019) are particularly susceptible to the impacts of particulate matter-induced depression, as previously demonstrated. Further stratified analyses were performed to explore changes across socioeconomic status, considering sex, age group, exercise, current smoking, current drinking, education attainment, marital status, social contact, self-reported health status, and private medical insurance.

Sensitivity analysis was conducted to assess the robustness of the findings. First, we conducted a generalized linear mixed model with a binomial distribution and logit link, mirroring the specifications of the GEE model, while incorporating the random intercept of each subject. Second, we treated depression as a continuous scale rather than a binary one, employing a linear mixed model to investigate the relationship between air pollutants and the continuous CES-D 10 scale. Third, this involved evaluating EVI as an alternative representation of greenness in the models and investigating the effects of a 6-month exposure to air pollution. Moreover, supplementary exposure models were included for other air pollutants (NO_2_ and O_3_) and analysed separately from the main models to assess whether particulate matter demonstrates an impact even in the presence of other air pollutants (Borroni *et al.*, 2022; Lim *et al.*, 2012). Variance inflation factors (VIFs) were assessed to detect multicollinearity among variables, with a threshold of VIFs <2.5 indicating acceptable levels.

All statistical analyses were performed using SAS version 9.4 (SAS Institute Inc.) and R version 4.1.3. The significance level was set at *p* <0.05 for all analyses.

## Results

Table 1 shows the descriptive characteristics of the variables in the baseline, the first wave of each participant, by the quantiles of NDVI. The number of participants was 7657, 1953, 1947, 1687 and 2070 in total, quantile 1–4, respectively. The mean score of CES-D 10 was 16.20 (SD: 5.24). Overall 1700 (22.20%) participants in the sample had a depressive symptom in the baseline. Table 2 shows the descriptive and correlation analysis table of air pollution and greenness levels (see also supplementary Table S1). Almost two thirds of Korea’s topography is composed of mountains (Choung *et al.*, 2004), and the result is reflected in the NDVI figures (mean: 0.50, SD: 0.11, Supplementary Figure S2, Supplementary Table S2).

**Table 1.** Descriptive characteristics of the variables in the baseline of participants (Mean (SD) or proportion)

		Quantiles of NDVI				
Variables	Total	Quantile 1 (0.0–0.41)	Quantile 2 (0.42–0.49)	Quantile 3 (0.50–0.57)	Quantile 4 (0.58–1.00)	p-value
No of participants	7657	1953	1947	1687	2070	
CES-D 10 score	16.20 ± 5.24	17.36 ± 5.79	16.4 ± 5.49	15.18 ± 4.52	15.75 ± 4.80	<.0001
Age (years)	68.13 ± 10.34	68.6 ± 10.45	67.75 ± 10.06	68.22 ± 10.43	68.23 ± 10.38	0.4588
Sex						0.2791
Female	4390 (57.33)	1147 (58.73)	1110 (57.01)	938 (55.6)	1195 (57.73)	
Male	3267 (42.67)	806 (41.27)	837 (42.99)	749 (44.4)	875 (42.27)	
Current smoking (%)						0.0266
No	6820 (89.07)	1761 (90.17)	1752 (89.98)	1476 (87.49)	1831 (88.45)	
Yes	837 (10.93)	192 (9.83)	195 (10.02)	211 (12.51)	239 (11.55)	
Current drinking (%)						0.0425
No	5051 (65.97)	1306 (66.87)	1277 (65.59)	1147 (67.99)	1321 (63.82)	
Yes	2606 (34.03)	647 (33.13)	670 (34.41)	540 (32.01)	749 (36.18)	
Education attainment (%)						<.0001
Primary school and below	3030 (39.57)	695 (35.59)	677 (34.77)	679 (40.25)	979 (47.29)	
Middle and high school	3638 (47.51)	985 (50.44)	988 (50.74)	774 (45.88)	891 (43.04)	
College and above	989 (12.92)	273 (13.98)	282 (14.48)	234 (13.87)	200 (9.66)	
Marital status (%)						0.0022
Single, divorced and widowed, married and not living with spouse	1857 (24.25)	524 (26.83)	430 (22.09)	385 (22.82)	518 (25.02)	
Married and living with spouse	5800 (75.75)	1429 (73.17)	1517 (77.91)	1302 (77.18)	1552 (74.98)	
Social contact (%)						<.0001
Every other month or less often	1191 (15.55)	436 (22.32)	357 (18.34)	167 (9.9)	231 (11.16)	
Once a month or more often and less than once a week	1758 (22.96)	415 (21.25)	480 (24.65)	378 (22.41)	485 (23.43)	
Once a week or more often	4708 (61.49)	1102 (56.43)	1110 (57.01)	1142 (67.69)	1354 (65.41)	
Self-reported health status (%)						<.0001
Normal or bad	4378 (57.18)	1197 (61.29)	1038 (53.31)	979 (58.03)	1164 (56.23)	
Good	3279 (42.82)	756 (38.71)	909 (46.69)	708 (41.97)	906 (43.77)	
Exercise (%)						<.0001
No	4980 (65.04)	1169 (59.86)	1290 (66.26)	1118 (66.27)	1403 (67.78)	
Yes	2677 (34.96)	784 (40.14)	657 (33.74)	569 (33.73)	667 (32.22)	
Private medical insurance (%)						0.0036
No medical insurance	4925 (64.32)	1220 (62.47)	1238 (63.59)	1147 (67.99)	1320 (63.77)	
Having medical insurance	2732 (35.68)	733 (37.53)	709 (36.41)	540 (32.01)	750 (36.23)	
Household net worth (7.59 USD)						<.0001
1st quintile (∼7000)	1547 (20.20)	370 (19.31)	376 (19.20)	283 (16.66)	518 (24.86)	
2nd quintile (7001–15000)	1729 (22.58)	331 (17.28)	419 (21.40)	433 (25.49)	546 (26.20)	
3rd quintile (15001–23000)	1342 (17.53)	294 (15.34)	338 (17.26)	328 (19.31)	382 (18.33)	
4th quintile (23001–39000)	1523 (19.89)	376 (19.62)	421 (21.50)	391 (23.01)	335 (16.07)	
5th quintile (39000∼)	1516 (19.80)	545 (28.44)	404 (20.63)	264 (15.54)	303 (14.54)	
Density of population (/km^2^)						<.0001
1st quintile (∼280.4)	1575 (20.57)	0 (0.00)	133 (6.79)	473 (27.84)	969 (46.50)	
2nd quintile (280.5–1125.5)	1452 (18.96)	70 (3.65)	405 (20.68)	295 (17.36)	682 (32.73)	
3rd quintile (1125.6–4745.7)	1607 (20.99)	335 (17.48)	364 (18.59)	542 (31.90)	366 (17.56)	
4th quintile (4745.8–10891.2)	1507 (19.68)	453 (23.64)	598 (30.54)	389 (22.90)	67 (3.21)	
5th quintile (10891.2∼)	1516 (19.80)	1058 (55.22)	458 (23.39)	0 (0.00)	0 (0.00)	
Number of beds in hospitals per 1,000 persons	14.84 ± 7.96	11.94 ± 6.9	14.61 ± 7.97	16.36 ± 7.43	16.49 ± 8.5	<.0001
Number of national basic livelihood beneficiaries	11,366.35 ± 6,838.58	12,385.5 ± 5,498.88	14,092.19 ± 7,450.37	10,334.5 ± 5,977.52	8,709.57 ± 6,851.41	<.0001
Independent rate of finance of local government	30.8 ± 13.52	38.08 ± 12.9	33.33 ± 15.21	26.61 ± 11.02	25.12 ± 10.06	<.0001
Proportion of basic pension beneficiaries	65.71 ± 9.76	59.03 ± 10.32	63.56 ± 8.27	67.68 ± 7.55	72.27 ± 7.05	<.0001

*Note*: Table 1 presents descriptive statistics of the study participants, including proportions, means and standard deviations, categorized by the baseline year (i.e., the year when participants initially entered the study, ranging from 2016 to 2020). ANOVA or chi-squared test p-value was presented to compare quantile groups of NDVI.

Abbreviation: CES-D, Center for Epidemiology Studies of Depression scale; PM_10_, particulate matter of 10 microns in diameter or smaller; PM_2.5_, particulate matter of 2.5 microns in diameter or smaller; NDVI, Normalized Difference Vegetation Index; SD, standard deviation.

**Table 2.** Descriptive and correlation table of air pollution and greenness averaged between 2016, 2018 and 2020

						Correlations			
	Mean	SD	Min	Median	Max	PM_10_	PM_2.5_	NDVI	EVI
PM_10_	38.92	6.51	25.25	39.44	56.21	1	–	–	–
PM_2.5_	22.01	3.63	13.43	22.19	32.62	.879***	1	–	–
NDVI	0.50	0.11	0.22	0.52	0.68	−.649***	−.501***	1	–
EVI	0.28	0.07	0.11	0.30	0.37	−.565***	−.377***	.973***	1

PM_10_, particulate matter of 10 microns in diameter or smaller; PM_2.5_, particulate matter of 2.5 microns in diameter or smaller; NDVI, Normalized Difference Vegetation Index; EVI, Enhanced Vegetation Index; SD, standard deviation.

*** *p* < 0.001

**Figure 1.**
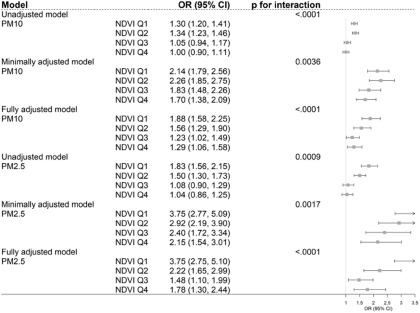
Associations between air pollution exposure (per 10 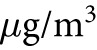 increase) and depression by NDVI quantiles.

*Note*: The OR was derived from the GEE model, with depression characterized by a CES-D 10 score of 20 or higher. Unadjusted model: adjusted for particulate matter (PM_10_ or PM_2.5_), NDVI quantiles, and interaction term of particulate matter and NDVI quantiles. Minimally adjusted model: adjusted for particulate matter (PM_10_ or PM_2.5_), NDVI quantiles, interaction term of particulate matter and NDVI quantiles, year, longitude, latitude and interaction term of longitude and latitude. Fully adjusted model: adjusted for particulate matter (PM_10_ or PM_2.5_), NDVI quantiles, interaction term of particulate matter and NDVI quantiles, year, longitude, latitude and interaction term of longitude and latitude, age, sex, current smoking, current drinking, education attainment, marital status, social contact, self-reported health status, exercise, year, private medical insurance, density of population quintiles, number of beds in hospitals per 1,000 persons, number of national basic livelihood beneficiaries, independent rate of finance of local government, and proportion of basic pension beneficiaries. The *p*-values for interactions were calculated from models that included interaction terms for each particulate matter and NDVI. PM_2.5_: particulate matter with an aerodynamic diameter ≤2.5 µm; PM_10_: particulate matter with an aerodynamic diameter ≤10 µm; NDVI: normalized difference vegetation index.

Figure 1 shows the minimally or fully adjusted GEE model results of air pollution effects by NDVI quantile level on CES-D 10 score. The ORs are the ORs of air pollution on depressive symptoms considering the NDVI quantile effect. In the fully adjusted model, the ORs for depression with a 10 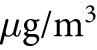 increment of PM_10_ were 1.88 (95% CI: 1.58, 2.25) in NDVI Q1 and 1.29 (95% CI: 1.06, 1.58) in NDVI Q4 (*p* for interaction < .0001). With a 10 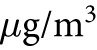 increment of PM_2.5_, the ORs for depression were 3.75 (95% CI: 2.75, 5.10) in NDVI Q1 and 1.78 (95% CI: 1.30, 2.44) in NDVI Q4 (*p* for interaction < .0001). The ORs in the minimally adjusted generalized estimation equation model were consistent with the ORs in the fully adjusted model. The ORs for depression showed a positive association with PM_10_ and PM_2.5_ over all NDVI quantiles, but compared to the ORs in NDVI Q1, the ORs significantly decreased in NDVI Q4.

Several sensitivity analyses were also performed. The generalized linear mixed models and linear mixed models were shown in Supplementary Table S3–4. Thus, instead of NDVI, EVI was added as a green effect and analysed with PM_10_ (OR: 1.92 [95% CI: 1.61, 2.29] in EVI Q1, 1.25 [1.02, 1.54] in EVI Q4, *p* for interaction < .0001) and PM_2.5_ (4.02 [2.96, 5.47] in EVI Q1, 1.53 [1.11, 2.12] in EVI Q4, *p* for interaction < .0001) (Supplementary Table S5). These findings indicate that the OR for particulate matter in EVI Q4 was lower than that in EVI Q1, a pattern consistent with the results of the main model depicted in Fig. 1. One year exposure to air pollution was performed in the main model, we also performed 6 months exposure to air pollution (Supplementary Table S6). Both PM_10_ and PM_2.5_ of the models showed the odd ratios in NDVI Q4 were significantly decreased compared to NDVI Q1. Additionally, the observed relationships between particulate matter and NDVI interaction with depression remained consistent even after controlling for other pollutants such as NO2 and O3, as demonstrated by the two-pollutant models (Supplementary Table S7).

**Figure 2.**
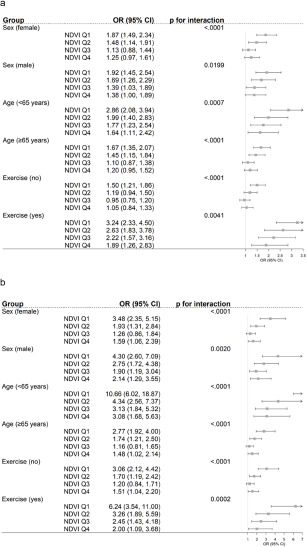
(a) Subgroup analysis of associations between air pollution (PM_10_) exposure (per 10 increase) and depression by NDVI quantiles. (b) Subgroup analysis of associations between air pollution (PM_2.5_) exposure (per 10 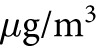 increase) and depression by NDVI quantiles.

*Note*: The OR was derived from the GEE model, with depression characterized by a CES-D 10 score of 20 or higher. The models were adjusted for PM_10_, NDVI quantiles, interaction term of PM_10_ and NDVI quantiles, year, longitude, latitude and interaction term of longitude and latitude, age, sex, current smoking, current drinking, education attainment, marital status, social contact, self-reported health status, exercise, year, private medical insurance, density of population quintiles, number of beds in hospitals per 1,000 persons, number of national basic livelihood beneficiaries, independent rate of finance of local government and proportion of basic pension beneficiaries, except for the subgroup variable itself in the model. The *p*-values for interactions were calculated from models that included interaction terms for PM_10_ and NDVI. PM_10_: particulate matter with an aerodynamic diameter ≤10 µm; NDVI: normalized difference vegetation index. *Note*: The OR was derived from the GEE model, with depression characterized by a CES-D 10 score of 20 or higher.The models were adjusted for PM_2.5_, NDVI quantiles, interaction term of PM_2.5_ and NDVI quantiles, year, longitude, latitude and interaction term of longitude and latitude, age, sex, current smoking, current drinking, education attainment, marital status, social contact, self-reported health status, exercise, year, private medical insurance, density of population quintiles, number of beds in hospitals per 1,000 persons, number of national basic livelihood beneficiaries, independent rate of finance of local government, and proportion of basic pension beneficiaries, except for the subgroup variable itself in the model. The *p*-values for interactions were calculated from models that included interaction terms for PM_2.5_ and NDVI. PM_2.5_: particulate matter with an aerodynamic diameter ≤2.5 µm; NDVI: normalized difference vegetation index.

Figure 2 shows the results of stratified analyses showing the estimated association (OR) between air pollution and depression by NDVI quantiles among separate subgroups such as sex, age group and exercise. In NDVI Q1, the ORs for depression per 10 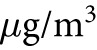 increase in PM_10_ were higher among the stratified groups compared to the ORs in NDVI Q4. The results of PM_2.5_ were consistent with those of PM_10_, and additional stratified analysis results are provided in Supplementary Table S8.

## Discussion

The association between greenness (NDVI) and air pollution in the KLoSA has been found to significantly alleviate the negative impacts of air pollution on depression. This finding particularly holds true for the highest quantile (Q4) of NDVI compared to the lowest quantile (Q1). PM_2.5_ exhibited higher ORs compared to PM_10_. Subgroup analysis results demonstrated beneficial effects of greenness in the relationship between particulate matter and depression.

This study examines the association of greenness with air pollution on depression, employing the CES-D 10 questionnaire as an outcome measure. The findings of this study align with previous studies that also employed a questionnaire or utilized antidepressant use as an outcome measure. The Yinzhou Cohort, a Chinese study that followed participants for five years, revealed decreased hazard ratios for depression per interquartile range increase in NDVI after adjusting for PM_2.5_ and PM_10_, respectively (Zhang *et al.*, 2022). Another study, a cross-sectional study based on the Dutch national health survey, demonstrated a significant association between greenness, antidepressant use, and PM_2.5_ exposure. The ORs for NDVI exposure and PM_2.5_ exposure were 0.96 (95% CI: 0.94, 0.98) and 1.01 (95% CI: 0.99, 1.03), respectively in the adjusted model accounting for two exposures (Klompmaker *et al.*, 2019). A study conducted in Guangzhou, utilizing a cross-sectional survey design, discovered a beneficial association between green spaces, such as street trees, and psychological well-being, which was mediated by PM_2.5_ levels (Wang *et al.*, 2020). A systematic review was conducted to assess the impact of residential greenness on the relationship between air pollution and various health outcomes, including mental health. However, this review found only a limited number of studies available for direct comparison with the present study (Son *et al.*, 2021).

Several studies have explored the relationship between greenness and depression, yielding noteworthy findings. In a cross-sectional study conducted in the United Kingdom, participants aged 37–73 years demonstrated an OR of 0.96 (95% CI: 0.93, 0.99) for depression, as assessed using a questionnaire on probable major depressive disorder experiences, in relation to NDVI (Sarkar *et al.*, 2018). An American Nurses’ Health Study, which followed a cohort of individuals aged 54–91 years, revealed that those residing in areas with the highest quintile of greenness within a 250-meter radius exhibited a lower risk of depression compared to those in the lowest quintile (HR: 0.87, 95% CI: 0.78, 0.98) (Banay *et al.*, 2019). A study conducted in Henan Province, China, employing the Patient Health Questionnaire-2, indicated a negative association between an interquartile range increase in NDVI and depression in a mixed model adjusted for covariates (−0.024, 95% CI: −0.041, −0.006) (Di *et al.*, 2020). However, contrary to previous findings, some studies conducted in the United States did not establish a significant association between greenness and depression (Pun *et al.*, 2018).

The reduction of particulate matter within green spaces has been proposed as a physical mechanism (Choi *et al.*, 2022), involving processes such as adsorption, absorption, blocking and sedimentation of particulate matter. The microstructure of trees provides an ideal surface for the attachment of particulate matter (Sgrigna *et al.*, 2015), while the leaves of tress have the capacity to absorb particulate matter (Liu *et al.*, 2016). The presence of green space creates turbulence in the surrounding air, which can lead to the reduction or obstruction of particulate matter (Bernatzky, 1983; Jin *et al.*, 2014; Vesala *et al.*, 2005). Particulate matter tends to settle on the leaves, stems, and branches of tress, highlighting the effectiveness of green spaces in reducing particulate matter levels (Janhäll, 2015). Beyond minimizing harm, greenness has been associated with various protective mechanisms that contribute to mental health. These mechanisms include the restoration and enhancement of cognitive capacities (Markevych *et al.*, 2017), and an increase in physical activity levels (James *et al.*, 2015). The biophila hypothesis, developed by Wilson, proposes a natural affinity between plants and people (White and Heerwagen, 1998; Wilson, 1984), and it has been frequently mentioned in studies investigating the relationship between greenness and mental health. The availability of green spaces has the potential to foster social cohesiveness and encourage social interactions through recreational or regular physical activities, which are strongly linked to mental well-being (Berkman *et al.*, 2014).

The group-specific analysis results uniformly indicated a consistent beneficial role of greenness in the association between particulate matter and depression. Previous studies into the association between greenness and depression have yielded significant that were sex-specific, appearing exclusively among males (Di *et al.*, 2020) or exclusively among the females (De Vries *et al.*, 2003; Sarkar *et al.*, 2018; Triguero-Mas *et al.*, 2015), as well as age-specific, with effects observed among participants aged over 65 years (Sarkar *et al.*, 2018) or those below 65 years (Di *et al.*, 2020; Wang *et al.*, 2019a).

Our study has several limitations. First, the assessment of people’s depression was reliant on self-questionnaires rather than utilizing formal diagnoses from medical professionals, which could lead to outcome misclassification. However, it is crucial to highlight that the CES-D 10, the depression score employed in our study, has been extensively validated and demonstrated strong reliability in previous research studies (Boey, 1999; Cole *et al.*, 2004; Malakouti *et al.*, 2015). Secondly, apart from the inclusion of air pollutants such as NO_2_, CO and O_3_, our analysis did not account for other conceivable environmental factors, such as noise pollution, which have the potential to influence the manifestation of depression (van den Bosch and Meyer-Lindenberg, 2019). Thirdly, it is important to acknowledge that people’s exposure to air pollution was estimated on a district (si/gun/gu) basis rather than an individual basis. We made the assumption that people residing within the same district would experience comparable levels of air pollution concentration and greenness level. Despite being second-level local authority areas within metropolitan cities and provinces in South Korea, districts are characterized by a median area size of approximately 397 km^2^, which is roughly 1.7 times larger than a ZIP code in the United States (233 km^2^). Consequently, we hypothesize that the effects of air pollution can be adequately captured in nationwide-scale environmental epidemiological studies (Di *et al.*, 2017; Lee *et al.*, 2021; Park *et al.*, 2023), despite the challenge of accurately reflecting individual-level exposures. However, this approximation may oversimplify the actual exposure variations experienced by people within the district boundaries. These limitations underscore the need for further research endeavours to address the aforementioned gaps and enhance the precision and comprehensiveness of our understanding of the complicated relationship between air pollution, environmental factors and depression.

Above these limitations, our study draws upon a high-quality longitudinal study conducted in Korea, characterized by extensive national coverage, specifically focusing on the elderly population across diverse regions. This study represents the pioneering investigation in Korea examining the association between greenness, particulate matters and depression in older individuals, while effectively adjusting for socioeconomic characteristics, social relationships and health-related variables. Given Korea’s rapid progression towards a super-aging society, the findings of this study hold substantial utility for addressing depressive conditions in the elderly population (Park *et al.*, 2014). This study could contribute to enhancing our comprehension of depression in elderly Koreans by elucidating the environmental factors that can either protect or exert detrimental effects. The findings can be instrumental in formulating policy implications for the betterment of this population. Furthermore, in addition to utilizing NDVI, this study also incorporates EVI as a measure of residential greenness in the supplementary results. The utilization of EVI, a vegetation index that accounts for seasonal and interannual variations in vegetation production with greater precision, further enhances the methodological robustness of the study (Leng *et al.*, 2022).

## Supporting information

Park et al. supplementary material Park et al. supplementary material

## Data Availability

The data used in this study are not publicly available due to restrictions imposed by the Korea Employment Information Service (KEIS) and AiMS-CREATE, from where the data were obtained under license. However, the authors can provide the data upon reasonable request and with permission from KEIS and AiMS-CREATE.

## Supplementary material

The supplementary material for this article can be found at https://doi.org/10.1017/S2045796024000684.
