# Supplementary material for: Association between air pollution (PM10, PM2.5), greenness and depression in older adults: a longitudinal study in South Korea
Source: Epidemiol Psychiatr Sci. 2024 Nov 27;33:e71. doi: 10.1017/S2045796024000684 (PMC11669799; doi:10.1017/S2045796024000684)
Supplement: Park et al. supplementary material [file S2045796024000684sup001.docx]

**[Supplementary Material]**

**Supplementary 1. Air Pollution Prediction Model**

Air pollution prediction models were developed by AiMS-CREATE network. For the full process and result of prediction model and more details can be found below:
<https://papers.ssrn.com/sol3/papers.cfm?abstract_id=4280944>

*1.1 Modeling procedures*

To establish the prediction model, predictor variables were collected from the google earth engine (GEE), Socioeconomic Data and Applications Center (SEDAC), and regional socioeconomic database as input variables, and each of the five air pollution concentrations (PM_2.5_, PM_10_, NO_2_, O_3_, and CO) were predicted as the outcome variables. Missing values in the predictor variables were replaced with values through the imputation process. Randomly selected 80% of the monitoring stations were used to train the models, and the remaining 20% of the monitoring stations were used to test the performance of the model. Each of the three machine learning models (i.e., random forest, light gradient boosting, and neural network model) was trained with 10-fold cross-validation (CV) in the training set to avoid overfitting problems. We validated the model performance using R^2^ and root mean squared error (RMSE) with three machine learning models and simple averages among models (ensemble model) in the test set. Finally, we predicted monthly basis air pollution concentrations with a 1 km × 1 km grid during 2002–2020 using the three machine learning-based models and ensemble models.

*1.2 Ensemble model*

To aggregate the predictions, we calculated the simple averages of each machine learning estimation.

$$\hat{Y}_{SAij}= \frac{\hat{Y}_{RFij}+ \hat{Y}_{GBij}+ \hat{Y}_{NNij}}{3}$$

$\hat{Y}_{RFij}$, $\hat{Y}_{GBij}$, 𝑎𝑛𝑑 $\hat{Y}_{NNij}$ are predicted air pollution concentrations from the random forest, light gradient boosting, and neural network, respectively, at location i at time j. 𝑌_𝑆𝐴𝑖𝑗_ is the simple averaged prediction values derived by averaging the three estimations at location i at time j. We also trained a generalized additive model (GAM) to consider the geographical variation of each of the three machine-learning estimations.

**Supplementary Figure 1.** Directed Acyclic Graph for the association between particulate matter (PM_10_, PM_2.5_) and depression.

**
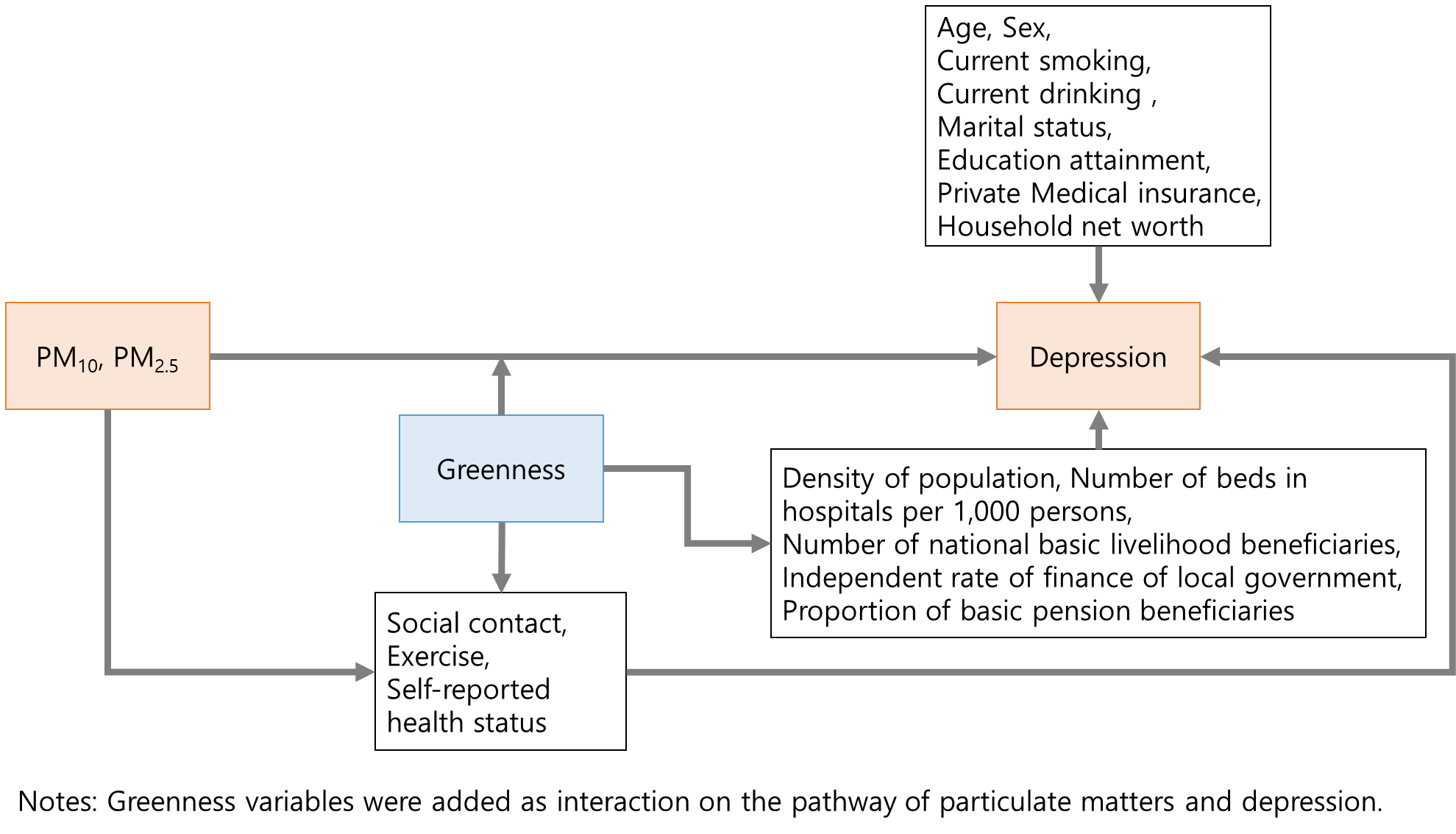
**

**Supplementary Figure 2.** Geographical distribution of NDVI level averaged between 2016, 2018 and 2020 in South Korea.


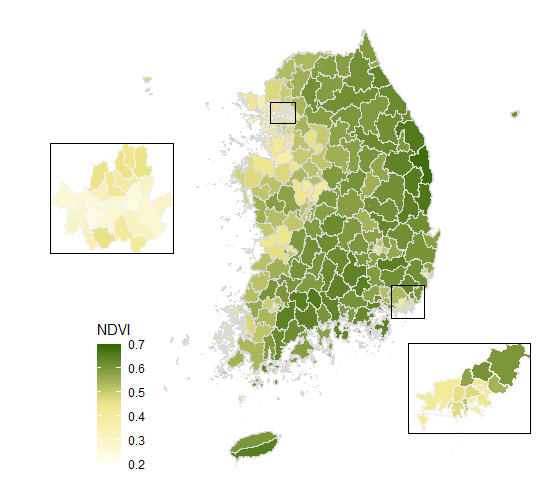


**Supplementary Table 1.** Descriptive table of greenness and air pollution by year.

| Year | Variables | Mean | SD | Min | Median | Max |
| --- | --- | --- | --- | --- | --- | --- |
| 2016 | NDVI | 0.51 | 0.11 | 0.22 | 0.53 | 0.67 |
|  | EVI | 0.28 | 0.07 | 0.11 | 0.3 | 0.37 |
|  | PM_10_ | 45.17 | 3.94 | 36.28 | 44.53 | 56.21 |
|  | PM_2.5_ | 25.48 | 2.16 | 20.65 | 25.32 | 32.62 |
|  | NO_2_ | 17.47 | 5.21 | 11.68 | 15.15 | 29.07 |
|  | CO | 0.53 | 0.03 | 0.47 | 0.52 | 0.62 |
|  | O_3_ | 41.83 | 2.67 | 34.04 | 42.83 | 46.17 |
| 2018 | NDVI | 0.51 | 0.11 | 0.23 | 0.53 | 0.68 |
|  | EVI | 0.28 | 0.07 | 0.11 | 0.31 | 0.38 |
|  | PM_10_ | 39.67 | 2.70 | 33.22 | 39.45 | 49.32 |
|  | PM_2.5_ | 22.39 | 1.65 | 17.86 | 22.24 | 27.72 |
|  | NO_2_ | 16.64 | 4.9 | 11.07 | 14.64 | 27.65 |
|  | CO | 0.51 | 0.03 | 0.47 | 0.51 | 0.61 |
|  | O_3_ | 41.84 | 3.01 | 33.83 | 42.84 | 46.57 |
| 2020 | NDVI | 0.49 | 0.11 | 0.23 | 0.51 | 0.67 |
|  | EVI | 0.27 | 0.06 | 0.11 | 0.29 | 0.36 |
|  | PM_10_ | 31.96 | 3.95 | 25.25 | 31.42 | 42.65 |
|  | PM_2.5_ | 18.19 | 2.28 | 13.43 | 18.33 | 23.61 |
|  | NO_2_ | 15.27 | 4.48 | 9.62 | 13.54 | 25.16 |
|  | CO | 0.51 | 0.03 | 0.46 | 0.50 | 0.60 |
|  | O_3_ | 42.82 | 2.76 | 35.77 | 43.62 | 46.85 |

**Supplementary Table 2.** Average concentrations of particulate matters and greennness during the study period (2016, 2018, 2020) and regional characteristics of 217 districts in South Korea.

| No | Province | District | PM_2.5_ | PM_10_ | NDVI | EVI | Longitude | Latitude | Population  density (/km^2^) |
| --- | --- | --- | --- | --- | --- | --- | --- | --- | --- |
| 1 | Seoul | Jongno-gu | 22.04 | 40.74 | 0.43 | 0.22 | 126.98 | 37.59 | 6680.47 |
| 2 | Seoul | Jung-gu | 22.7 | 39.92 | 0.28 | 0.14 | 127 | 37.56 | 12985.55 |
| 3 | Seoul | Yongsan-gu | 23.01 | 40.91 | 0.28 | 0.14 | 126.98 | 37.53 | 10891.2 |
| 4 | Seoul | Eunpyung-gu | 22.06 | 40.25 | 0.45 | 0.23 | 126.93 | 37.62 | 16760.45 |
| 5 | Seoul | Seongdong-gu | 22.89 | 42.76 | 0.24 | 0.12 | 127.04 | 37.55 | 17720.08 |
| 6 | Seoul | Gwangjin-gu | 23.17 | 42.48 | 0.28 | 0.13 | 127.09 | 37.54 | 21421.67 |
| 7 | Seoul | Dongdaemun-gu | 22.77 | 41.05 | 0.25 | 0.13 | 127.06 | 37.58 | 25475.77 |
| 8 | Seoul | Jungnang-gu | 22.47 | 41.16 | 0.32 | 0.16 | 127.1 | 37.6 | 22558.82 |
| 9 | Seoul | Seongbuk-gu | 22.06 | 41.58 | 0.39 | 0.2 | 127.02 | 37.6 | 19124.7 |
| 10 | Seoul | Nowon-gu | 21.9 | 40.24 | 0.45 | 0.25 | 127.08 | 37.65 | 16538.18 |
| 11 | Seoul | Gangbuk-gu | 21.67 | 38.91 | 0.45 | 0.24 | 127.01 | 37.64 | 14267.6 |
| 12 | Seoul | Gangseo-gu | 24.06 | 46.01 | 0.27 | 0.14 | 126.83 | 37.56 | 13980.47 |
| 13 | Seoul | Dobong-gu | 21.73 | 39.23 | 0.45 | 0.25 | 127.04 | 37.67 | 17228.85 |
| 14 | Seoul | Seodaemun-gu | 22.58 | 40.66 | 0.35 | 0.17 | 126.94 | 37.58 | 17803.75 |
| 15 | Seoul | Mapo-gu | 23.07 | 41.13 | 0.26 | 0.13 | 126.91 | 37.56 | 16142.07 |
| 16 | Seoul | Yangcheon-gu | 23.95 | 42.93 | 0.29 | 0.14 | 126.86 | 37.52 | 28127.75 |
| 17 | Seoul | Geumcheon-gu | 23.36 | 40.74 | 0.35 | 0.18 | 126.9 | 37.46 | 18416.32 |
| 18 | Seoul | Guro-gu | 23.79 | 43.03 | 0.32 | 0.16 | 126.86 | 37.49 | 21086 |
| 19 | Seoul | Yeongdeungpo-gu | 23.64 | 42.99 | 0.23 | 0.11 | 126.91 | 37.52 | 15667.05 |
| 20 | Seoul | Dongjak-gu | 23.34 | 40.98 | 0.26 | 0.12 | 126.95 | 37.5 | 24733.05 |
| 21 | Seoul | Gangnam-gu | 22.69 | 41.08 | 0.32 | 0.17 | 127.07 | 37.49 | 14409.32 |
| 22 | Seoul | Gwanak-gu | 22.84 | 41.15 | 0.45 | 0.22 | 126.95 | 37.46 | 17457.33 |
| 23 | Seoul | Seocho-gu | 22.86 | 42.76 | 0.41 | 0.22 | 127.03 | 37.47 | 9411.62 |
| 24 | Seoul | Songpa-gu | 22.97 | 41.38 | 0.27 | 0.15 | 127.12 | 37.5 | 19712.22 |
| 25 | Seoul | Gangdong-gu | 23.78 | 43.09 | 0.31 | 0.16 | 127.15 | 37.55 | 19290.93 |
| 26 | Busan | Jung-gu | 23.11 | 38.59 | 0.29 | 0.12 | 129.03 | 35.1 | 16636.77 |
| 27 | Busan | Seo-gu | 21.44 | 37.25 | 0.51 | 0.24 | 129.02 | 35.1 | 8505.38 |
| 28 | Busan | Dong-gu | 20.79 | 37.29 | 0.41 | 0.2 | 129.05 | 35.13 | 9783.98 |
| 29 | Busan | Nam-gu | 20.26 | 37.66 | 0.45 | 0.21 | 129.1 | 35.12 | 10736.28 |
| 30 | Busan | Yeongdo-gu | 19.73 | 36.86 | 0.4 | 0.18 | 129.07 | 35.08 | 9504.48 |
| 31 | Busan | Busanjin-gu | 20.94 | 36.87 | 0.47 | 0.25 | 129.05 | 35.16 | 13040.82 |
| 32 | Busan | Sasang-gu | 22.71 | 39.73 | 0.46 | 0.23 | 128.99 | 35.15 | 6766.97 |
| 33 | Busan | Dongrae-gu | 21.94 | 38.47 | 0.44 | 0.22 | 129.08 | 35.2 | 16640.98 |
| 34 | Busan | Gangseo-gu | 22.48 | 38.73 | 0.4 | 0.23 | 128.91 | 35.15 | 444.25 |
| 35 | Busan | Haeundae-gu | 20.19 | 35.82 | 0.54 | 0.28 | 129.16 | 35.19 | 8243.47 |
| 36 | Busan | Buk-gu | 21.04 | 36.21 | 0.57 | 0.29 | 129.03 | 35.23 | 7868.27 |
| 37 | Busan | Saha-gu | 23.36 | 39.02 | 0.47 | 0.21 | 128.98 | 35.09 | 8278.42 |
| 38 | Busan | Geumjeong-gu | 20.97 | 37.02 | 0.6 | 0.32 | 129.09 | 35.26 | 3843.05 |
| 39 | Busan | Yeonje-gu | 21.65 | 38.82 | 0.41 | 0.21 | 129.09 | 35.18 | 17378.4 |
| 40 | Busan | Suyeong-gu | 21.96 | 37.78 | 0.42 | 0.21 | 129.11 | 35.16 | 17410.45 |
| 41 | Busan | Gijang-gun | 20 | 34.67 | 0.59 | 0.33 | 129.2 | 35.3 | 619.68 |
| 42 | Daegu | Jung-gu | 21.32 | 39.67 | 0.23 | 0.11 | 128.6 | 35.86 | 11049.92 |
| 43 | Daegu | Buk-gu | 21.44 | 39.47 | 0.52 | 0.28 | 128.58 | 35.93 | 4731.05 |
| 44 | Daegu | Dalseo-gu | 20.95 | 37.71 | 0.42 | 0.23 | 128.53 | 35.83 | 9689.77 |
| 45 | Daegu | Dong-gu | 21.18 | 38 | 0.59 | 0.32 | 128.69 | 35.93 | 1899.33 |
| 46 | Daegu | Seo-gu | 23.11 | 41.66 | 0.33 | 0.19 | 128.55 | 35.87 | 12218.63 |
| 47 | Daegu | Nam-gu | 21.06 | 37.8 | 0.42 | 0.21 | 128.59 | 35.83 | 9412.47 |
| 48 | Daegu | Suseong-gu | 20.75 | 37.41 | 0.5 | 0.26 | 128.66 | 35.83 | 5966.02 |
| 49 | Daegu | Dalseong-gun | 20.05 | 37.29 | 0.57 | 0.32 | 128.51 | 35.73 | 447.3 |
| 50 | Incheon | Bupyeong-gu | 23.05 | 42.25 | 0.33 | 0.18 | 126.72 | 37.49 | 17392.55 |
| 51 | Incheon | Jung-gu | 22.04 | 39.02 | 0.33 | 0.18 | 126.47 | 37.47 | 808.8 |
| 52 | Incheon | Dong-gu | 23.2 | 45.03 | 0.23 | 0.11 | 126.64 | 37.48 | 10386.03 |
| 53 | Incheon | Yeonsu-gu | 22.05 | 41.73 | 0.35 | 0.19 | 126.65 | 37.37 | 6335.65 |
| 54 | Incheon | Namdong-gu | 23.14 | 44.01 | 0.34 | 0.2 | 126.73 | 37.43 | 9010.9 |
| 55 | Incheon | Gyeyang-gu | 24.2 | 43.71 | 0.39 | 0.22 | 126.74 | 37.55 | 7440.58 |
| 56 | Incheon | Seo-gu | 23.03 | 43.2 | 0.34 | 0.19 | 126.65 | 37.56 | 4221.4 |
| 57 | Incheon | Ganghwa-gun | 22.67 | 38.52 | 0.44 | 0.26 | 126.45 | 37.7 | 163.3 |
| 58 | Incheon | Nam-gu | 23.47 | 43.78 | 0.26 | 0.14 | 126.67 | 37.45 | 16600.6 |
| 59 | Gwangju | Dong-gu | 20.7 | 37.07 | 0.59 | 0.32 | 126.95 | 35.11 | 2067.15 |
| 60 | Gwangju | Seo-gu | 20.56 | 37.41 | 0.4 | 0.23 | 126.85 | 35.13 | 6539.07 |
| 61 | Gwangju | Buk-gu | 20.74 | 36.8 | 0.51 | 0.28 | 126.93 | 35.19 | 3697.3 |
| 62 | Gwangju | Nam-gu | 21.92 | 37.65 | 0.46 | 0.27 | 126.86 | 35.09 | 3591.65 |
| 63 | Gwangju | Gwangsan-gu | 21.59 | 35.97 | 0.49 | 0.29 | 126.76 | 35.16 | 1760.65 |
| 64 | Daejeon | Dong-gu | 21.44 | 38.49 | 0.57 | 0.28 | 127.48 | 36.32 | 1797.67 |
| 65 | Daejeon | Seo-gu | 20.51 | 38.18 | 0.5 | 0.27 | 127.35 | 36.28 | 5188.62 |
| 66 | Daejeon | Jung-gu | 20.56 | 38.53 | 0.53 | 0.28 | 127.41 | 36.28 | 4196.15 |
| 67 | Daejeon | Yuseong-gu | 21.26 | 39.06 | 0.52 | 0.29 | 127.34 | 36.37 | 2072.82 |
| 68 | Daejeon | Daedeok-gu | 23.35 | 42.67 | 0.5 | 0.26 | 127.44 | 36.41 | 2929.3 |
| 69 | Ulsan | Jung-gu | 20.78 | 37.73 | 0.49 | 0.27 | 129.31 | 35.57 | 6423.3 |
| 70 | Ulsan | Buk-gu | 20.17 | 36.56 | 0.57 | 0.34 | 129.38 | 35.61 | 1194.23 |
| 71 | Ulsan | Nam-gu | 20.86 | 38.02 | 0.38 | 0.21 | 129.33 | 35.51 | 4738.75 |
| 72 | Ulsan | Dong-gu | 19.33 | 36.95 | 0.49 | 0.25 | 129.43 | 35.52 | 4867.43 |
| 73 | Ulsan | Ulju-gun | 20.28 | 36.83 | 0.61 | 0.35 | 129.19 | 35.54 | 280.45 |
| 74 | Gyeonggi | Ansan-si | 23.95 | 44.34 | 0.42 | 0.23 | 126.84 | 37.32 | 4697.68 |
| 75 | Gyeonggi | Uijeongbu-si | 22.89 | 43.02 | 0.5 | 0.27 | 127.07 | 37.73 | 5302.12 |
| 76 | Gyeonggi | Goyang-si | 24.6 | 44.91 | 0.37 | 0.21 | 126.8 | 37.67 | 3728.7 |
| 77 | Gyeonggi | Yangju-si | 23.61 | 42.88 | 0.5 | 0.29 | 127 | 37.81 | 651.25 |
| 78 | Gyeonggi | Guri-si | 24.15 | 44.64 | 0.4 | 0.22 | 127.13 | 37.6 | 5725 |
| 79 | Gyeonggi | Anyang-si | 24.05 | 44.71 | 0.44 | 0.24 | 126.94 | 37.4 | 10349.8 |
| 80 | Gyeonggi | Gimpo-si | 26.64 | 45.3 | 0.36 | 0.21 | 126.63 | 37.68 | 1151.1 |
| 81 | Gyeonggi | Bucheon-si | 25.39 | 47.33 | 0.34 | 0.18 | 126.79 | 37.5 | 16100.35 |
| 82 | Gyeonggi | Paju-si | 25.17 | 44.49 | 0.48 | 0.28 | 126.81 | 37.85 | 604.5 |
| 83 | Gyeonggi | Suwon-si | 23.45 | 43.81 | 0.37 | 0.2 | 127.02 | 37.28 | 9514.58 |
| 84 | Gyeonggi | Seongnam-si | 22.45 | 43.47 | 0.46 | 0.26 | 127.13 | 37.41 | 6891.2 |
| 85 | Gyeonggi | Gwangju-si | 25.3 | 45.18 | 0.54 | 0.31 | 127.3 | 37.4 | 683.17 |
| 86 | Gyeonggi | Yeoncheon-gun | 22.87 | 38.11 | 0.53 | 0.32 | 127.03 | 38.09 | 67.35 |
| 87 | Gyeonggi | Gunpo-si | 24.13 | 45.43 | 0.47 | 0.26 | 126.92 | 37.34 | 7877.22 |
| 88 | Gyeonggi | Uiwang-si | 24.23 | 43.25 | 0.5 | 0.28 | 126.99 | 37.36 | 2892.65 |
| 89 | Gyeonggi | Gwangmyeong-si | 24.91 | 45.82 | 0.41 | 0.23 | 126.87 | 37.44 | 9073.7 |
| 90 | Gyeonggi | Pyeongtaek-si | 28.09 | 48.86 | 0.37 | 0.23 | 127.02 | 37.03 | 977.47 |
| 91 | Gyeonggi | Dongducheon-si | 23.32 | 40.9 | 0.54 | 0.31 | 127.08 | 37.91 | 1018.88 |
| 92 | Gyeonggi | Siheung-si | 26.51 | 48.7 | 0.38 | 0.23 | 126.79 | 37.39 | 2948.35 |
| 93 | Gyeonggi | Gwacheon-si | 23.51 | 44.75 | 0.51 | 0.28 | 127.01 | 37.43 | 1935.33 |
| 94 | Gyeonggi | Namyangju-si | 23.83 | 41.92 | 0.54 | 0.31 | 127.25 | 37.66 | 1363.82 |
| 95 | Gyeonggi | Osan-si | 25.1 | 45.09 | 0.36 | 0.21 | 127.05 | 37.16 | 4772.02 |
| 96 | Gyeonggi | Hanam-si | 21.95 | 43.9 | 0.45 | 0.25 | 127.21 | 37.52 | 1731.43 |
| 97 | Gyeonggi | Yongin-si | 25.46 | 44.77 | 0.48 | 0.27 | 127.15 | 37.27 | 1601.23 |
| 98 | Gyeonggi | Icheon-si | 26.75 | 45.32 | 0.45 | 0.28 | 127.48 | 37.21 | 446.25 |
| 99 | Gyeonggi | Anseong-si | 25.66 | 43.93 | 0.5 | 0.3 | 127.31 | 37.03 | 328.12 |
| 100 | Gyeonggi | Hwaseong-si | 27.23 | 47.59 | 0.41 | 0.24 | 126.88 | 37.16 | 808.85 |
| 101 | Gyeonggi | Pocheon-si | 23.34 | 40.81 | 0.55 | 0.32 | 127.25 | 37.97 | 189.1 |
| 102 | Gyeonggi | Gapyeong-gun | 21.77 | 37.96 | 0.61 | 0.34 | 127.45 | 37.82 | 72.48 |
| 103 | Gyeonggi | Yangpyeong-gun | 22.98 | 40.58 | 0.59 | 0.33 | 127.58 | 37.52 | 119.68 |
| 104 | Gyeonggi | Yeoju-si | 25.1 | 42.82 | 0.5 | 0.3 | 127.62 | 37.3 | 181.08 |
| 105 | Gangwon | Cheorwon-gun | 22.72 | 38.82 | 0.54 | 0.31 | 127.39 | 38.23 | 53.97 |
| 106 | Gangwon | Yeongwol-gun | 22.51 | 39.68 | 0.62 | 0.33 | 128.5 | 37.2 | 35.62 |
| 107 | Gangwon | Chuncheon-si | 21.37 | 39.69 | 0.59 | 0.32 | 127.74 | 37.89 | 247.08 |
| 108 | Gangwon | Wonju-si | 23.51 | 40.55 | 0.57 | 0.32 | 127.93 | 37.31 | 376.82 |
| 109 | Gangwon | Gangneung-si | 19.38 | 34.76 | 0.62 | 0.33 | 128.83 | 37.71 | 207.68 |
| 110 | Gangwon | Donghae-si | 18.23 | 33.25 | 0.61 | 0.32 | 129.06 | 37.5 | 524.07 |
| 111 | Gangwon | Taebaek-si | 20.65 | 36.13 | 0.58 | 0.31 | 128.98 | 37.17 | 159.98 |
| 112 | Gangwon | Sokcho-si | 20.16 | 36.92 | 0.56 | 0.29 | 128.52 | 38.17 | 783.65 |
| 113 | Gangwon | Samcheok-si | 18.25 | 33.8 | 0.65 | 0.34 | 129.12 | 37.27 | 60.45 |
| 114 | Gangwon | Hongcheon-gun | 22.45 | 39.35 | 0.62 | 0.34 | 128.08 | 37.74 | 38.55 |
| 115 | Gangwon | Hoengseong-gun | 22.56 | 39.19 | 0.6 | 0.34 | 128.08 | 37.51 | 45.28 |
| 116 | Gangwon | Pyeongchang-gun | 21.79 | 37.73 | 0.59 | 0.33 | 128.49 | 37.55 | 29.75 |
| 117 | Gangwon | Jeongseon-gun | 20.59 | 36.83 | 0.61 | 0.33 | 128.74 | 37.38 | 32.42 |
| 118 | Gangwon | Hwacheon-gun | 21.24 | 36.88 | 0.59 | 0.34 | 127.68 | 38.12 | 28.52 |
| 119 | Gangwon | Goseong-gun | 19.93 | 36.41 | 0.59 | 0.34 | 128.43 | 38.36 | 45.67 |
| 120 | Gangwon | Yangyang-gun | 20.2 | 36.69 | 0.63 | 0.34 | 128.6 | 38 | 43.82 |
| 121 | Chungcheongbuk-do | Chungju-si | 23.12 | 40.67 | 0.55 | 0.31 | 127.9 | 37.01 | 211.77 |
| 122 | Chungcheongbuk-do | Cheongju-si | 24.72 | 41.78 | 0.44 | 0.26 | 127.47 | 36.64 | 885.73 |
| 123 | Chungcheongbuk-do | Jecheon-si | 22.21 | 38.64 | 0.58 | 0.32 | 128.14 | 37.06 | 155.07 |
| 124 | Chungcheongbuk-do | Boeun-gun | 22.75 | 39.11 | 0.59 | 0.33 | 127.73 | 36.49 | 58.83 |
| 125 | Chungcheongbuk-do | Okcheon-gun | 23.24 | 38.25 | 0.58 | 0.32 | 127.66 | 36.32 | 98.25 |
| 126 | Chungcheongbuk-do | Yeongdong-gun | 22.05 | 38.32 | 0.62 | 0.35 | 127.82 | 36.16 | 59.9 |
| 127 | Chungcheongbuk-do | Jincheon-gun | 24.79 | 41.91 | 0.52 | 0.3 | 127.44 | 36.87 | 161.32 |
| 128 | Chungcheongbuk-do | Umseong-gun | 25.17 | 42.83 | 0.51 | 0.3 | 127.62 | 36.97 | 181.83 |
| 129 | Chungcheongbuk-do | Jeungpyeong-gun | 24.7 | 40.99 | 0.49 | 0.29 | 127.61 | 36.78 | 430.98 |
| 130 | Chungcheongbuk-do | Danyang-gun | 17.99 | 30.59 | 0.6 | 0.32 | 128.39 | 36.99 | 39.75 |
| 131 | Chungcheongbuk-do | Gwoisan-gun | 21.31 | 36 | 0.59 | 0.32 | 127.83 | 36.77 | 45.35 |
| 132 | Chungcheongnam-do | Seocheon-gun | 23.25 | 39.74 | 0.5 | 0.29 | 126.71 | 36.1 | 161.23 |
| 133 | Chungcheongnam-do | Cheonan-si | 25.71 | 43.21 | 0.49 | 0.29 | 127.21 | 36.8 | 934.48 |
| 134 | Chungcheongnam-do | Asan-si | 25.97 | 44.47 | 0.47 | 0.28 | 126.98 | 36.8 | 533.85 |
| 135 | Chungcheongnam-do | Gongju-si | 23.22 | 39.96 | 0.57 | 0.33 | 127.08 | 36.48 | 131.73 |
| 136 | Chungcheongnam-do | Nonsan-si | 22.35 | 40.43 | 0.5 | 0.3 | 127.16 | 36.19 | 225.98 |
| 137 | Chungcheongnam-do | Boryung-si | 22.78 | 38.82 | 0.55 | 0.31 | 126.62 | 36.32 | 184.13 |
| 138 | Chungcheongnam-do | Seosan-si | 22.35 | 40.5 | 0.47 | 0.27 | 126.47 | 36.78 | 224.77 |
| 139 | Chungcheongnam-do | Gyeryong-si | 22.21 | 37.87 | 0.57 | 0.33 | 127.24 | 36.29 | 687.27 |
| 140 | Chungcheongnam-do | Geumsan-gun | 21.84 | 38.39 | 0.59 | 0.33 | 127.48 | 36.12 | 95.87 |
| 141 | Chungcheongnam-do | Buyeo-gun | 24.23 | 39.38 | 0.52 | 0.31 | 126.86 | 36.24 | 115.55 |
| 142 | Chungcheongnam-do | Cheongyang-gun | 24.05 | 39.44 | 0.58 | 0.33 | 126.86 | 36.43 | 67.48 |
| 143 | Chungcheongnam-do | Hongseong-gun | 24.66 | 39.59 | 0.51 | 0.3 | 126.63 | 36.57 | 207.45 |
| 144 | Chungcheongnam-do | Yesan-gun | 24.77 | 41.29 | 0.51 | 0.3 | 126.79 | 36.67 | 155.88 |
| 145 | Chungcheongnam-do | Taean-gun | 22.29 | 38.57 | 0.49 | 0.26 | 126.26 | 36.77 | 122.4 |
| 146 | Chungcheongnam-do | Dangjin-si | 24.78 | 44.64 | 0.45 | 0.26 | 126.66 | 36.89 | 228.53 |
| 147 | Jeollabuk-do | Gunsan-si | 24.24 | 40.91 | 0.43 | 0.25 | 126.76 | 35.96 | 702.2 |
| 148 | Jeollabuk-do | Jeonju-si | 24.26 | 41.31 | 0.46 | 0.26 | 127.12 | 35.83 | 3161.1 |
| 149 | Jeollabuk-do | Wanju-gun | 23.93 | 39.9 | 0.59 | 0.34 | 127.23 | 35.92 | 109.43 |
| 150 | Jeollabuk-do | Iksan-si | 26.24 | 43.15 | 0.44 | 0.28 | 126.99 | 36.02 | 601.9 |
| 151 | Jeollabuk-do | Jeongeup-si | 23.43 | 38.34 | 0.55 | 0.32 | 126.91 | 35.6 | 169.95 |
| 152 | Jeollabuk-do | Namwon-si | 21.17 | 36.9 | 0.59 | 0.33 | 127.44 | 35.42 | 114.22 |
| 153 | Jeollabuk-do | Gimje-si | 25.05 | 42.09 | 0.46 | 0.29 | 126.91 | 35.8 | 166.13 |
| 154 | Jeollabuk-do | Jinan-gun | 22.19 | 37.96 | 0.61 | 0.34 | 127.43 | 35.83 | 33.97 |
| 155 | Jeollabuk-do | Muju-gun | 21.82 | 36.92 | 0.62 | 0.35 | 127.72 | 35.94 | 40.13 |
| 156 | Jeollabuk-do | Imshil-gun | 22.65 | 38.69 | 0.6 | 0.34 | 127.24 | 35.6 | 50.52 |
| 157 | Jeollabuk-do | Sunchang-gun | 23.04 | 38.46 | 0.6 | 0.34 | 127.09 | 35.43 | 60.62 |
| 158 | Jeollabuk-do | Buan-gun | 23.8 | 39.85 | 0.52 | 0.3 | 126.66 | 35.67 | 117.8 |
| 159 | Jeollanam-do | Mokpo-si | 19.03 | 35.44 | 0.39 | 0.2 | 126.41 | 34.81 | 4745.75 |
| 160 | Jeollanam-do | Yeosu-si | 17.46 | 31.92 | 0.58 | 0.31 | 127.65 | 34.77 | 574.63 |
| 161 | Jeollanam-do | Suncheon-si | 19.08 | 33.02 | 0.64 | 0.36 | 127.39 | 34.99 | 303.35 |
| 162 | Jeollanam-do | Naju-si | 21.02 | 34.68 | 0.53 | 0.32 | 126.72 | 34.99 | 152.65 |
| 163 | Jeollanam-do | Gwangyang-si | 18.65 | 34.96 | 0.6 | 0.34 | 127.65 | 35.03 | 331.23 |
| 164 | Jeollanam-do | Damyang-gun | 22.53 | 37.77 | 0.59 | 0.34 | 127 | 35.29 | 104.1 |
| 165 | Jeollanam-do | Gokseong-gun | 20.63 | 35.63 | 0.64 | 0.35 | 127.27 | 35.21 | 56.3 |
| 166 | Jeollanam-do | Goheung-gun | 19.45 | 33.69 | 0.58 | 0.33 | 127.33 | 34.62 | 88.05 |
| 167 | Jeollanam-do | Boseong-gun | 21.65 | 35.57 | 0.61 | 0.35 | 127.16 | 34.81 | 69.57 |
| 168 | Jeollanam-do | Jangheung-gun | 20.04 | 35.08 | 0.62 | 0.36 | 126.92 | 34.67 | 68.45 |
| 169 | Jeollanam-do | Gangjin-gun | 21.65 | 36.48 | 0.59 | 0.35 | 126.77 | 34.62 | 79.32 |
| 170 | Jeollanam-do | Haenam-gun | 21.84 | 35.21 | 0.53 | 0.32 | 126.53 | 34.54 | 76.17 |
| 171 | Jeollanam-do | Yeongam-gun | 21.83 | 34.93 | 0.51 | 0.3 | 126.64 | 34.8 | 97.53 |
| 172 | Jeollanam-do | Muan-gun | 22.03 | 35.86 | 0.47 | 0.28 | 126.43 | 34.95 | 176.88 |
| 173 | Jeollanam-do | Hampyeong-gun | 22.26 | 36.22 | 0.56 | 0.34 | 126.54 | 35.11 | 90.03 |
| 174 | Jeollanam-do | Yeonggwang-gun | 22.69 | 37.45 | 0.52 | 0.31 | 126.46 | 35.27 | 119.47 |
| 175 | Jeollanam-do | Jangseong-gun | 22.04 | 37.18 | 0.6 | 0.34 | 126.77 | 35.33 | 89.23 |
| 176 | Jeollanam-do | Jindo-gun | 21.29 | 35.12 | 0.54 | 0.32 | 126.25 | 34.46 | 74.63 |
| 177 | Jeollanam-do | Hwansun-gun | 19.77 | 33.7 | 0.63 | 0.35 | 127.04 | 35.01 | 85.4 |
| 178 | Jeollanam-do | Wando-gun | 19.83 | 33.65 | 0.62 | 0.32 | 126.7 | 34.34 | 134.75 |
| 179 | Jeollanam-do | Gurye-gun | 20.47 | 35.74 | 0.65 | 0.37 | 127.51 | 35.23 | 61.48 |
| 180 | Gyeongsangbuk-do | Gumi-si | 21.41 | 39.57 | 0.54 | 0.31 | 128.36 | 36.17 | 679.8 |
| 181 | Gyeongsangbuk-do | Pohang-si | 19.74 | 35.91 | 0.6 | 0.34 | 129.34 | 36.06 | 458.97 |
| 182 | Gyeongsangbuk-do | Gyeongju-si | 20.95 | 35.94 | 0.6 | 0.35 | 129.24 | 35.82 | 198 |
| 183 | Gyeongsangbuk-do | Gimcheon-si | 22.02 | 38.82 | 0.59 | 0.34 | 128.08 | 36.06 | 136.22 |
| 184 | Gyeongsangbuk-do | Andong-si | 20.48 | 36 | 0.62 | 0.34 | 128.78 | 36.58 | 110.78 |
| 185 | Gyeongsangbuk-do | Yeongju-si | 22.87 | 39.28 | 0.57 | 0.33 | 128.6 | 36.87 | 166.87 |
| 186 | Gyeongsangbuk-do | Yeongcheon-si | 20.99 | 37.05 | 0.62 | 0.35 | 128.95 | 36.01 | 110.3 |
| 187 | Gyeongsangbuk-do | Sangju-si | 22.07 | 38.05 | 0.58 | 0.33 | 128.07 | 36.43 | 82.25 |
| 188 | Gyeongsangbuk-do | Mungyeong-si | 21.06 | 36.71 | 0.6 | 0.34 | 128.15 | 36.69 | 83.13 |
| 189 | Gyeongsangbuk-do | Gyeongsan-si | 22.22 | 37.82 | 0.57 | 0.33 | 128.81 | 35.83 | 610.32 |
| 190 | Gyeongsangbuk-do | Uiseong-gun | 20.78 | 36.41 | 0.62 | 0.35 | 128.62 | 36.36 | 47.45 |
| 191 | Gyeongsangbuk-do | Yeongyang-gun | 19.93 | 36.26 | 0.64 | 0.35 | 129.15 | 36.69 | 22.18 |
| 192 | Gyeongsangbuk-do | Yeongdeok-gun | 19.64 | 34.23 | 0.66 | 0.36 | 129.32 | 36.48 | 53.75 |
| 193 | Gyeongsangbuk-do | Cheongdo-gun | 21.14 | 35.24 | 0.64 | 0.36 | 128.79 | 35.67 | 63.25 |
| 194 | Gyeongsangbuk-do | Goreong-gun | 19.69 | 35.15 | 0.6 | 0.33 | 128.31 | 35.73 | 91.07 |
| 195 | Gyeongsangbuk-do | Chilgok-gun | 21.47 | 38.95 | 0.58 | 0.33 | 128.47 | 36.01 | 269.02 |
| 196 | Gyeongsangbuk-do | Yecheon-gun | 21.74 | 36.58 | 0.55 | 0.32 | 128.43 | 36.65 | 69.08 |
| 197 | Gyeongsangbuk-do | Bonghwa-gun | 19.58 | 35.92 | 0.64 | 0.34 | 128.92 | 36.93 | 28.2 |
| 198 | Gyeongsangbuk-do | Uljin-gun | 19.56 | 35.32 | 0.68 | 0.35 | 129.32 | 36.9 | 52.48 |
| 199 | Gyeongsangnam-do | Yangsan-si | 21.59 | 37.82 | 0.62 | 0.35 | 129.04 | 35.4 | 593.07 |
| 200 | Gyeongsangnam-do | Gimhae-si | 20.67 | 36.65 | 0.53 | 0.3 | 128.85 | 35.27 | 1125.53 |
| 201 | Gyeongsangnam-do | Changwon-si | 20.36 | 37.57 | 0.58 | 0.32 | 128.66 | 35.27 | 1445.53 |
| 202 | Gyeongsangnam-do | Jinju-si | 20 | 36.26 | 0.6 | 0.34 | 128.13 | 35.2 | 477.18 |
| 203 | Gyeongsangnam-do | Tongyeong-si | 18.89 | 34.73 | 0.62 | 0.3 | 128.4 | 34.9 | 581.9 |
| 204 | Gyeongsangnam-do | Sacheon-si | 18.99 | 34.65 | 0.59 | 0.33 | 128.04 | 35.05 | 289.82 |
| 205 | Gyeongsangnam-do | Miryang-si | 20.76 | 36.64 | 0.61 | 0.35 | 128.79 | 35.5 | 135.75 |
| 206 | Gyeongsangnam-do | Geoje-si | 19.4 | 35.21 | 0.63 | 0.33 | 128.63 | 34.86 | 610.43 |
| 207 | Gyeongsangnam-do | Uiryeong-gun | 21.07 | 35.71 | 0.64 | 0.36 | 128.28 | 35.39 | 60.62 |
| 208 | Gyeongsangnam-do | Haman-gun | 21.04 | 36.98 | 0.57 | 0.32 | 128.43 | 35.29 | 163.55 |
| 209 | Gyeongsangnam-do | Changnyeong-gun | 21.35 | 35.98 | 0.59 | 0.33 | 128.5 | 35.51 | 118.95 |
| 210 | Gyeongsangnam-do | Goseong-gun | 19.88 | 37.07 | 0.62 | 0.35 | 128.29 | 35.01 | 108.32 |
| 211 | Gyeongsangnam-do | Namhae-gun | 18.89 | 36.04 | 0.63 | 0.35 | 127.93 | 34.81 | 132.07 |
| 212 | Gyeongsangnam-do | Hadong-gun | 19.89 | 35.64 | 0.63 | 0.36 | 127.78 | 35.14 | 75.02 |
| 213 | Gyeongsangnam-do | Sancheong-gun | 20.47 | 36.32 | 0.63 | 0.36 | 127.89 | 35.37 | 45.15 |
| 214 | Gyeongsangnam-do | Geochang-gun | 21.02 | 38.34 | 0.62 | 0.35 | 127.91 | 35.73 | 78.67 |
| 215 | Gyeongsangnam-do | Hapcheon-gun | 20.38 | 36.8 | 0.63 | 0.35 | 128.14 | 35.57 | 50.63 |
| 216 | Gyeongsangnam-do | Hamyang-gun | 20.28 | 35.38 | 0.62 | 0.35 | 127.72 | 35.55 | 55.98 |
| 217 | Sejong | Sejong | 24.11 | 42.39 | 0.51 | 0.3 | 127.22 | 36.53 | 363.66 |

**Supplementary Table 3.** Associations between air pollution exposure (per 10 $\mu g/m^{3}$ increase) and depression by NDVI quantiles by linear mixed models.

| Variables | Model | Estimate | 95% CI | | P for interaction |
| --- | --- | --- | --- | --- | --- |
| PM_10_ (per 10$\mu g/m^{3}$) | Unadjusted model |  |  |  | <.0001 |
|  | NDVI Q1 | 0.79 | 0.60 | 0.98 |  |
|  | NDVI Q2 | 0.35 | 0.18 | 0.52 |  |
|  | NDVI Q3 | 0.25 | 0.08 | 0.42 |  |
|  | NDVI Q4 | -0.13 | -0.31 | 0.05 |  |
| PM_10_ (per 10$\mu g/m^{3}$) | Minimally adjusted model |  |  |  | <.0001 |
|  | NDVI Q1 | 1.49 | 1.16 | 1.81 |  |
|  | NDVI Q2 | 1.02 | 0.68 | 1.35 |  |
|  | NDVI Q3 | 0.84 | 0.52 | 1.17 |  |
|  | NDVI Q4 | 0.53 | 0.21 | 0.84 |  |
| PM_10_ (per 10$\mu g/m^{3}$) | Fully adjusted model |  |  |  | <.0001 |
|  | NDVI Q1 | 1.15 | 0.83 | 1.47 |  |
|  | NDVI Q2 | 0.43 | 0.09 | 0.77 |  |
|  | NDVI Q3 | 0.36 | 0.04 | 0.68 |  |
|  | NDVI Q4 | 0.17 | -0.15 | 0.49 |  |
| PM2.5 (per 10$\mu g/m^{3}$) | Unadjusted model |  |  |  | <.0001 |
|  | NDVI Q1 | 1.72 | 1.35 | 2.09 |  |
|  | NDVI Q2 | 0.47 | 0.18 | 0.76 |  |
|  | NDVI Q3 | 0.48 | 0.19 | 0.77 |  |
|  | NDVI Q4 | -0.16 | -0.46 | 0.15 |  |
| PM2.5 (per 10$\mu g/m^{3}$) | Minimally adjusted model |  |  |  | <.0001 |
|  | NDVI Q1 | 2.86 | 2.28 | 3.44 |  |
|  | NDVI Q2 | 1.48 | 0.97 | 1.99 |  |
|  | NDVI Q3 | 1.37 | 0.87 | 1.88 |  |
|  | NDVI Q4 | 0.91 | 0.40 | 1.42 |  |
| PM2.5 (per 10$\mu g/m^{3}$) | Fully adjusted model |  |  |  | <.0001 |
|  | NDVI Q1 | 2.49 | 1.92 | 3.06 |  |
|  | NDVI Q2 | 0.85 | 0.33 | 1.37 |  |
|  | NDVI Q3 | 0.80 | 0.30 | 1.30 |  |
|  | NDVI Q4 | 0.55 | 0.03 | 1.07 |  |

Note: The estimates were derived from the linear mixed models.

Unadjusted model: adjusted for particulate matter (PM_10_ or PM_2.5_), NDVI quantiles, and interaction term of particulate matter and NDVI quantiles.

Minimally adjusted model: adjusted for particulate matter (PM_10_ or PM_2.5_), NDVI quantiles, interaction term of particulate matter and NDVI quantiles, year, longitude, latitude and interaction term of longitude and latitude.

Fully adjusted model: adjusted for particulate matter (PM_10_ or PM_2.5_), NDVI quantiles, interaction term of particulate matter and NDVI quantiles, year, longitude, latitude and interaction term of longitude and latitude, age, sex, current smoking, current drinking, education attainment, marital status, social contact, self-reported health status, exercise, year, private medical insurance, density of population quintiles, number of beds in hospitals per 1,000 persons, number of national basic livelihood beneficiaries, independent rate of finance of local government, and proportion of basic pension beneficiaries.

The p-values for interactions were calculated from models that included interaction terms for each particulate matter and NDVI.

PM_2.5_: particulate matter with an aerodynamic diameter ≤ 2.5 µm; PM_10_: particulate matter with an aerodynamic diameter ≤ 10 µm; NDVI: normalized difference vegetation index.

**Supplementary Table 4.** Associations between air pollution exposure (per 10 $\mu g/m^{3}$ increase) and depression by NDVI quantiles by generalized linear mixed models.

| Variables | Model | OR | 95% CI | | P for interaction |
| --- | --- | --- | --- | --- | --- |
| PM_10_ (per 10$\mu g/m^{3}$) | Unadjusted model |  |  |  | <.0001 |
|  | NDVI Q1 | 1.49 | 1.35 | 1.64 |  |
|  | NDVI Q2 | 1.48 | 1.34 | 1.64 |  |
|  | NDVI Q3 | 1.04 | 0.92 | 1.17 |  |
|  | NDVI Q4 | 1.00 | 0.89 | 1.13 |  |
| PM_10_ (per 10$\mu g/m^{3}$) | Minimally adjusted model |  |  |  | <.0001 |
|  | NDVI Q1 | 2.80 | 2.39 | 3.28 |  |
|  | NDVI Q2 | 2.83 | 2.39 | 3.36 |  |
|  | NDVI Q3 | 1.87 | 1.57 | 2.24 |  |
|  | NDVI Q4 | 1.87 | 1.56 | 2.24 |  |
| PM_10_ (per 10$\mu g/m^{3}$) | Fully adjusted model |  |  |  | <.0001 |
|  | NDVI Q1 | 2.10 | 1.75 | 2.52 |  |
|  | NDVI Q2 | 1.63 | 1.33 | 2.00 |  |
|  | NDVI Q3 | 1.26 | 1.03 | 1.54 |  |
|  | NDVI Q4 | 1.31 | 1.07 | 1.60 |  |
| PM_2.5_ (per 10$\mu g/m^{3}$) | Unadjusted model |  |  |  | <.0001 |
|  | NDVI Q1 | 2.41 | 2.00 | 2.90 |  |
|  | NDVI Q2 | 1.67 | 1.41 | 1.98 |  |
|  | NDVI Q3 | 1.10 | 0.90 | 1.34 |  |
|  | NDVI Q4 | 0.99 | 0.80 | 1.21 |  |
| PM_2.5_ (per 10$\mu g/m^{3}$) | Minimally adjusted model |  |  |  | <.0001 |
|  | NDVI Q1 | 4.76 | 3.65 | 6.21 |  |
|  | NDVI Q2 | 3.14 | 2.47 | 4.00 |  |
|  | NDVI Q3 | 1.89 | 1.45 | 2.47 |  |
|  | NDVI Q4 | 1.87 | 1.42 | 2.47 |  |
| PM_2.5_ (per 10$\mu g/m^{3}$) | Fully adjusted model |  |  |  | <.0001 |
|  | NDVI Q1 | 4.37 | 3.21 | 5.94 |  |
|  | NDVI Q2 | 2.23 | 1.67 | 2.99 |  |
|  | NDVI Q3 | 1.51 | 1.12 | 2.04 |  |
|  | NDVI Q4 | 1.67 | 1.22 | 2.27 |  |

Note: We estimated the odds ratios from the generalized linear mixed model (GLMM) with a binomial distribution, logit link, and random intercept for each subject. Depression was defined as having a CES-D 10 score of 20 points or over.

Unadjusted model: adjusted for particulate matter (PM_10_ or PM_2.5_), NDVI quantiles, and interaction term of particulate matter and NDVI quantiles.

Minimally adjusted model: adjusted for particulate matter (PM_10_ or PM_2.5_), NDVI quantiles, interaction term of particulate matter and NDVI quantiles, year, longitude, latitude and interaction term of longitude and latitude.

Fully adjusted model: adjusted for particulate matter (PM_10_ or PM_2.5_), NDVI quantiles, interaction term of particulate matter and NDVI quantiles, year, longitude, latitude and interaction term of longitude and latitude, age, sex, current smoking, current drinking, education attainment, marital status, social contact, self-reported health status, exercise, year, private medical insurance, density of population quintiles, number of beds in hospitals per 1,000 persons, number of national basic livelihood beneficiaries, independent rate of finance of local government, and proportion of basic pension beneficiaries.

The p-values for interactions were calculated from models that included interaction terms for each particulate matter and NDVI.

PM_2.5_: particulate matter with an aerodynamic diameter ≤ 2.5 µm; PM_10_: particulate matter with an aerodynamic diameter ≤ 10 µm; NDVI: normalized difference vegetation index.

**Supplementary Table 5.** Associations between air pollution exposure (per 10 $\mu g/m^{3}$ increase) and depression by EVI quantiles.

| Variables | Model | OR | 95% CI | | P for interaction |
| --- | --- | --- | --- | --- | --- |
| PM_10_ (per 10$\mu g/m^{3}$) | Fully adjusted model |  |  |  | <.0001 |
|  | EVI Q1 | 1.92 | 1.61 | 2.29 |  |
|  | EVI Q2 | 1.50 | 1.23 | 1.83 |  |
|  | EVI Q3 | 1.56 | 1.30 | 1.88 |  |
|  | EVI Q4 | 1.25 | 1.02 | 1.54 |  |
| PM_2.5_ (per 10$\mu g/m^{3}$) | Fully adjusted model |  |  |  | <.0001 |
|  | EVI Q1 | 4.02 | 2.96 | 5.47 |  |
|  | EVI Q2 | 1.94 | 1.43 | 2.65 |  |
|  | EVI Q3 | 2.04 | 1.55 | 2.68 |  |
|  | EVI Q4 | 1.53 | 1.11 | 2.12 |  |

Note: The odds ratio was derived from the generalized estimating equation (GEE) model, with depression characterized by a CES-D 10 score of 20 or higher.

The models were adjusted for particulate matter (PM_10_ or PM_2.5_), EVI quantiles, interaction term of particulate matter and EVI quantiles, year, longitude, latitude and interaction term of longitude and latitude, age, sex, current smoking, current drinking, education attainment, marital status, social contact, self-reported health status, exercise, year, private medical insurance, density of population quintiles, number of beds in hospitals per 1,000 persons, number of national basic livelihood beneficiaries, independent rate of finance of local government, and proportion of basic pension beneficiaries.

The p-values for interactions were calculated from models that included interaction terms for particulate matter and EVI .

PM_10_: particulate matter with an aerodynamic diameter ≤ 10 µm; PM_2.5_: particulate matter with an aerodynamic diameter ≤ 2.5 µm; EVI: enhanced vegetation index.

**Supplementary Table 6.** Associations between 6 months exposure to air pollution exposure (per 10 $\mu g/m^{3}$ increase) and depression by NDVI quantiles.

| Variables | Model | OR | 95% CI | | P for interaction |
| --- | --- | --- | --- | --- | --- |
| PM_10_ (per 10$\mu g/m^{3}$) | Fully adjusted model |  |  |  | <.0001 |
|  | NDVI Q1 | 1.30 | 1.14 | 1.49 |  |
|  | NDVI Q2 | 1.16 | 0.99 | 1.36 |  |
|  | NDVI Q3 | 0.83 | 0.70 | 0.98 |  |
|  | NDVI Q4 | 0.83 | 0.70 | 0.99 |  |
| PM_2.5_ (per 10$\mu g/m^{3}$) | Fully adjusted model |  |  |  | <.0001 |
|  | NDVI Q1 | 2.34 | 1.78 | 3.07 |  |
|  | NDVI Q2 | 1.70 | 1.28 | 2.26 |  |
|  | NDVI Q3 | 0.91 | 0.68 | 1.22 |  |
|  | NDVI Q4 | 1.04 | 0.76 | 1.40 |  |

Note: The odds ratio was derived from the generalized estimating equation (GEE) model, with depression characterized by a CES-D 10 score of 20 or higher.

The models were adjusted for particulate matter (PM_10_ or PM_2.5_), NDVI quantiles, interaction term of particulate matter and NDVI quantiles, year, longitude, latitude and interaction term of longitude and latitude, age, sex, current smoking, current drinking, education attainment, marital status, social contact, self-reported health status, exercise, year, private medical insurance, density of population quintiles, number of beds in hospitals per 1,000 persons, number of national basic livelihood beneficiaries, independent rate of finance of local government, and proportion of basic pension beneficiaries.

The p-values for interactions were calculated from models that included interaction terms for particulate matter and NDVI.

PM_10_: particulate matter with an aerodynamic diameter ≤ 10 µm; PM_2.5_: particulate matter with an aerodynamic diameter ≤ 2.5 µm; NDVI: normalized difference vegetation index.

**Supplementary Table 7.** Associations between air pollution exposure (per 10 $\mu g/m^{3}$ increase) plus NO_2_ and O_3_, and depression by NDVI quantiles.

| Variables | Model | OR | 95% CI | | P for interaction |
| --- | --- | --- | --- | --- | --- |
| PM_10_ (per 10$\mu g/m^{3}$)  plus NO_2_ | Fully adjusted model |  |  |  | <.0001 |
|  | NDVI Q1 | 1.89 | 1.58 | 2.25 |  |
|  | NDVI Q2 | 1.56 | 1.29 | 1.89 |  |
|  | NDVI Q3 | 1.21 | 1.00 | 1.47 |  |
|  | NDVI Q4 | 1.27 | 1.04 | 1.55 |  |
| PM_2.5_ (per 10$\mu g/m^{3}$)  plus NO_2_ | Fully adjusted model |  |  |  | <.0001 |
|  | NDVI Q1 | 3.76 | 2.76 | 5.13 |  |
|  | NDVI Q2 | 2.23 | 1.65 | 3.02 |  |
|  | NDVI Q3 | 1.49 | 1.10 | 2.03 |  |
|  | NDVI Q4 | 1.80 | 1.29 | 2.50 |  |
| PM_10_ (per 10$\mu g/m^{3}$) plus O_3_ | Fully adjusted model |  |  |  | <.0001 |
|  | NDVI Q1 | 1.79 | 1.50 | 2.13 |  |
|  | NDVI Q2 | 1.50 | 1.24 | 1.82 |  |
|  | NDVI Q3 | 1.15 | 0.95 | 1.40 |  |
|  | NDVI Q4 | 1.24 | 1.02 | 1.52 |  |
| PM_2.5_ (per 10$\mu g/m^{3}$)  plus O_3_ | Fully adjusted model |  |  |  | <.0001 |
|  | NDVI Q1 | 3.51 | 2.58 | 4.79 |  |
|  | NDVI Q2 | 2.12 | 1.58 | 2.86 |  |
|  | NDVI Q3 | 1.39 | 1.03 | 1.87 |  |
|  | NDVI Q4 | 1.73 | 1.26 | 2.38 |  |

Note: The odds ratio was derived from the generalized estimating equation (GEE) model, with depression characterized by a CES-D 10 score of 20 or higher.

The models were adjusted for particulate matter (PM_10_ or PM_2.5_), NDVI quantiles, interaction term of particulate matter and NDVI quantiles, year, longitude, latitude and interaction term of longitude and latitude, age, sex, current smoking, current drinking, education attainment, marital status, social contact, self-reported health status, exercise, year, private medical insurance, density of population quintiles, number of beds in hospitals per 1,000 persons, number of national basic livelihood beneficiaries, independent rate of finance of local government, and proportion of basic pension beneficiaries, plus the air pollution (NO_2_, O_3_).

The p-values for interactions were calculated from models that included interaction terms for particulate matter and NDVI.

PM_10_: particulate matter with an aerodynamic diameter ≤ 10 µm; PM_2.5_: particulate matter with an aerodynamic diameter ≤ 2.5 µm; NDVI: normalized difference vegetation index; NO_2_: nitrogen dioxide; O_3_: ozone.

Supplementary Table 8. Subgroup analysis of associations between air pollution exposure (per 10 $\mu g/m^{3}$ increase) and depression by NDVI quantiles.

| Subgroup |  | PM10 | | | P for interaction | PM2.5 | | | P for interaction |
| --- | --- | --- | --- | --- | --- | --- | --- | --- | --- |
|  | NDVI | OR | 95% CI | |  | OR | 95% CI | |  |
| Current smoking |  |  |  |  |  |  |  |  |  |
| No |  |  |  |  | <.0001 |  |  |  | <.0001 |
|  | NDVI Q1 | 1.78 | 1.48 | 2.14 |  | 3.43 | 2.49 | 4.73 |  |
|  | NDVI Q2 | 1.51 | 1.23 | 1.85 |  | 2.13 | 1.56 | 2.89 |  |
|  | NDVI Q3 | 1.11 | 0.91 | 1.36 |  | 1.29 | 0.95 | 1.76 |  |
|  | NDVI Q4 | 1.25 | 1.02 | 1.53 |  | 1.77 | 1.27 | 2.45 |  |
| Yes |  |  |  |  | 0.4407 |  |  |  | 0.1253 |
|  | NDVI Q1 | 1.92 | 0.99 | 3.75 |  | 5.30 | 1.59 | 17.70 |  |
|  | NDVI Q2 | 1.31 | 0.62 | 2.78 |  | 1.88 | 0.60 | 5.87 |  |
|  | NDVI Q3 | 1.83 | 0.91 | 3.68 |  | 3.70 | 1.19 | 11.49 |  |
|  | NDVI Q4 | 1.31 | 0.59 | 2.92 |  | 1.60 | 0.43 | 6.01 |  |
| Current drinking |  |  |  |  |  |  |  |  |  |
| No |  |  |  |  | <.0001 |  |  |  | <.0001 |
|  | NDVI Q1 | 1.56 | 1.27 | 1.92 |  | 2.46 | 1.72 | 3.53 |  |
|  | NDVI Q2 | 1.32 | 1.05 | 1.66 |  | 1.61 | 1.13 | 2.30 |  |
|  | NDVI Q3 | 1.06 | 0.84 | 1.32 |  | 1.14 | 0.81 | 1.61 |  |
|  | NDVI Q4 | 1.08 | 0.86 | 1.37 |  | 1.26 | 0.87 | 1.83 |  |
| Yes |  |  |  |  | 0.0008 |  |  |  | <.0001 |
|  | NDVI Q1 | 2.75 | 1.94 | 3.90 |  | 10.31 | 5.63 | 18.87 |  |
|  | NDVI Q2 | 2.16 | 1.50 | 3.10 |  | 4.30 | 2.51 | 7.37 |  |
|  | NDVI Q3 | 1.48 | 1.02 | 2.15 |  | 2.36 | 1.32 | 4.21 |  |
|  | NDVI Q4 | 1.90 | 1.30 | 2.78 |  | 4.08 | 2.23 | 7.46 |  |
| Education attainment |  |  |  |  |  |  |  |  |  |
| Primary school and below |  |  |  |  | <.0001 |  |  |  | <.0001 |
|  | NDVI Q1 | 1.54 | 1.18 | 2.01 |  | 2.46 | 1.56 | 3.86 |  |
|  | NDVI Q2 | 1.21 | 0.90 | 1.63 |  | 1.18 | 0.76 | 1.85 |  |
|  | NDVI Q3 | 0.86 | 0.64 | 1.14 |  | 0.78 | 0.51 | 1.20 |  |
|  | NDVI Q4 | 0.97 | 0.74 | 1.27 |  | 1.04 | 0.67 | 1.61 |  |
| Middle and high school |  |  |  |  | 0.0204 |  |  |  | 0.005 |
|  | NDVI Q1 | 1.88 | 1.44 | 2.44 |  | 4.35 | 2.72 | 6.97 |  |
|  | NDVI Q2 | 1.56 | 1.17 | 2.07 |  | 2.84 | 1.83 | 4.41 |  |
|  | NDVI Q3 | 1.30 | 0.97 | 1.75 |  | 2.01 | 1.26 | 3.19 |  |
|  | NDVI Q4 | 1.45 | 1.04 | 2.02 |  | 2.51 | 1.49 | 4.23 |  |
| College and above |  |  |  |  | 0.4043 |  |  |  | 0.1608 |
|  | NDVI Q1 | 2.87 | 1.58 | 5.22 |  | 9.09 | 3.21 | 25.76 |  |
|  | NDVI Q2 | 3.03 | 1.61 | 5.73 |  | 7.58 | 2.84 | 20.21 |  |
|  | NDVI Q3 | 2.80 | 1.51 | 5.20 |  | 5.18 | 2.07 | 12.98 |  |
|  | NDVI Q4 | 1.74 | 0.84 | 3.59 |  | 2.66 | 0.85 | 8.26 |  |
| Marital status |  |  |  |  |  |  |  |  |  |
| Single, divorced, and widowed, Married and not living with spouse |  |  |  |  | 0.0078 |  |  |  | 0.0008 |
|  | NDVI Q1 | 1.40 | 1.02 | 1.94 |  | 1.95 | 1.12 | 3.38 |  |
|  | NDVI Q2 | 1.03 | 0.71 | 1.51 |  | 0.85 | 0.49 | 1.50 |  |
|  | NDVI Q3 | 0.91 | 0.64 | 1.29 |  | 0.81 | 0.47 | 1.40 |  |
|  | NDVI Q4 | 1.06 | 0.75 | 1.50 |  | 1.04 | 0.60 | 1.80 |  |
| Married and living with spouse |  |  |  |  |  |  |  |  |  |
|  | NDVI Q1 |  |  |  | <.0001 |  |  |  | <.0001 |
|  | NDVI Q2 | 2.06 | 1.66 | 2.55 |  | 4.96 | 3.40 | 7.22 |  |
|  | NDVI Q3 | 1.78 | 1.42 | 2.24 |  | 3.16 | 2.23 | 4.50 |  |
|  | NDVI Q4 | 1.30 | 1.03 | 1.64 |  | 1.80 | 1.26 | 2.57 |  |
| Social contact |  |  |  |  |  |  |  |  |  |
| Every other month or less often |  |  |  |  | 0.8691 |  |  |  | 0.3002 |
|  | NDVI Q1 | 2.97 | 2.13 | 4.14 |  | 7.72 | 4.41 | 13.54 |  |
|  | NDVI Q2 | 2.81 | 1.93 | 4.08 |  | 5.63 | 3.19 | 9.94 |  |
|  | NDVI Q3 | 2.61 | 1.77 | 3.84 |  | 4.65 | 2.58 | 8.39 |  |
|  | NDVI Q4 | 2.94 | 2.00 | 4.32 |  | 6.89 | 3.68 | 12.91 |  |
| Once a month or more often and less than once a week |  |  |  |  | 0.0004 |  |  |  | 0.0007 |
|  | NDVI Q1 | 2.40 | 1.62 | 3.56 |  | 4.95 | 2.54 | 9.65 |  |
|  | NDVI Q2 | 2.41 | 1.57 | 3.70 |  | 3.34 | 1.82 | 6.15 |  |
|  | NDVI Q3 | 1.38 | 0.91 | 2.11 |  | 1.60 | 0.86 | 2.99 |  |
|  | NDVI Q4 | 1.46 | 0.95 | 2.24 |  | 1.85 | 0.98 | 3.47 |  |
| Once a week or more often |  |  |  |  | <.0001 |  |  |  | <.0001 |
|  | NDVI Q1 | 1.16 | 0.89 | 1.50 |  | 1.78 | 1.11 | 2.85 |  |
|  | NDVI Q2 | 0.78 | 0.57 | 1.06 |  | 0.82 | 0.51 | 1.31 |  |
|  | NDVI Q3 | 0.67 | 0.50 | 0.89 |  | 0.60 | 0.39 | 0.94 |  |
|  | NDVI Q4 | 0.71 | 0.53 | 0.95 |  | 0.65 | 0.40 | 1.06 |  |
| Self-reported health status |  |  |  |  |  |  |  |  |  |
| Normal or bad |  |  |  |  | <.0001 |  |  |  | <.0001 |
|  | NDVI Q1 | 1.82 | 1.46 | 2.26 |  | 2.75 | 1.89 | 4.01 |  |
|  | NDVI Q2 | 1.55 | 1.21 | 1.98 |  | 1.69 | 1.16 | 2.47 |  |
|  | NDVI Q3 | 1.12 | 0.88 | 1.42 |  | 1.13 | 0.78 | 1.62 |  |
|  | NDVI Q4 | 1.17 | 0.92 | 1.49 |  | 1.32 | 0.91 | 1.93 |  |
| Good |  |  |  |  | 0.0547 |  |  |  | <.0001 |
|  | NDVI Q1 | 2.44 | 1.79 | 3.31 |  | 9.64 | 5.73 | 16.20 |  |
|  | NDVI Q2 | 1.98 | 1.42 | 2.74 |  | 4.87 | 2.99 | 7.94 |  |
|  | NDVI Q3 | 1.76 | 1.25 | 2.48 |  | 3.37 | 2.00 | 5.70 |  |
|  | NDVI Q4 | 1.75 | 1.20 | 2.56 |  | 3.58 | 1.96 | 6.56 |  |
| Private medical insurance |  |  |  |  |  |  |  |  |  |
| No |  |  |  |  | <.0001 |  |  |  | <.0001 |
|  | NDVI Q1 | 1.63 | 1.33 | 1.99 |  | 2.79 | 1.97 | 3.97 |  |
|  | NDVI Q2 | 1.34 | 1.07 | 1.68 |  | 1.66 | 1.18 | 2.35 |  |
|  | NDVI Q3 | 1.01 | 0.81 | 1.26 |  | 1.06 | 0.76 | 1.47 |  |
|  | NDVI Q4 | 1.08 | 0.86 | 1.35 |  | 1.27 | 0.89 | 1.83 |  |
| Yes |  |  |  |  | 0.3063 |  |  |  | 0.0533 |
|  | NDVI Q1 | 3.44 | 2.37 | 5.00 |  | 10.90 | 5.67 | 20.97 |  |
|  | NDVI Q2 | 3.00 | 2.01 | 4.48 |  | 6.15 | 3.42 | 11.05 |  |
|  | NDVI Q3 | 2.79 | 1.86 | 4.18 |  | 5.56 | 2.93 | 10.56 |  |
|  | NDVI Q4 | 2.53 | 1.64 | 3.89 |  | 5.10 | 2.67 | 9.77 |  |

Note: The odds ratio was derived from the generalized estimating equation (GEE) model, with depression characterized by a CES-D 10 score of 20 or higher.

The models were adjusted for particulate matter (PM_10_ or PM_2.5_), NDVI quantiles, interaction term of particulate matter and NDVI quantiles, year, longitude, latitude and interaction term of longitude and latitude, age, sex, current smoking, current drinking, education attainment, marital status, social contact, self-reported health status, exercise, year, private medical insurance, density of population quintiles, number of beds in hospitals per 1,000 persons, number of national basic livelihood beneficiaries, independent rate of finance of local government, and proportion of basic pension beneficiaries, except for the subgroup variable itself in the model.

The p-values for interactions were calculated from models that included interaction terms for particulate matter and NDVI.

PM_10_: particulate matter with an aerodynamic diameter ≤ 10 µm; PM_2.5_: particulate matter with an aerodynamic diameter ≤ 2.5 µm; NDVI: normalized difference vegetation index.
